# Supplementary material for: Antibacterial macrocyclic peptides reveal a distinct mode of BamA inhibition
Source: Nat Commun. 2025 Apr 10;16:3395. doi: 10.1038/s41467-025-58086-w (PMC11986105; doi:10.1038/s41467-025-58086-w)
Supplement: Supplementary file 1 — Supplementary Information [file 41467_2025_58086_MOESM1_ESM.pdf]

Supplementary Information for:

Antibacterial macrocyclic peptides reveal a novel mode of BamA inhibition

Morgan E. Walker[1]*, Wei Zhu[2]*, Janine H. Peterson[3]*, Hao Wang[1], Jon Patteson[1], Aileen Soriano[2], Han Zhang[1], Todd Mayhood[2], Yan Hou[2], Samaneh Mesbahi-Vasey[1], Meigang Gu[4], John Frost[2], Jun Lu[1], Jennifer Johnston[2], Christopher Hipolito[2], Songnian Lin[2], Ronald E. Painter[1], Daniel Klein[1], Abbas Walji[1], Adam Weinglass[2], Terri M. Kelly[2], Adrian Saldanha[2], Jeffrey Schubert[1], Harris D. Bernstein[3]†, Scott S. Walker[1]†

[1]Merck & Co., Inc., West Point, PA, USA

[2]Merck & Co., Inc., Rahway, NJ, USA

[3]Genetics and Biochemistry Branch, National Institute of Diabetes and Digestive and Kidney Diseases, National Institutes of Health, Bethesda, MD 20892, USA

[4]Evotec Ltd., Abingdon, Oxfordshire OX14 4RZ, UK

* Contributed equally

† Corresponding authors

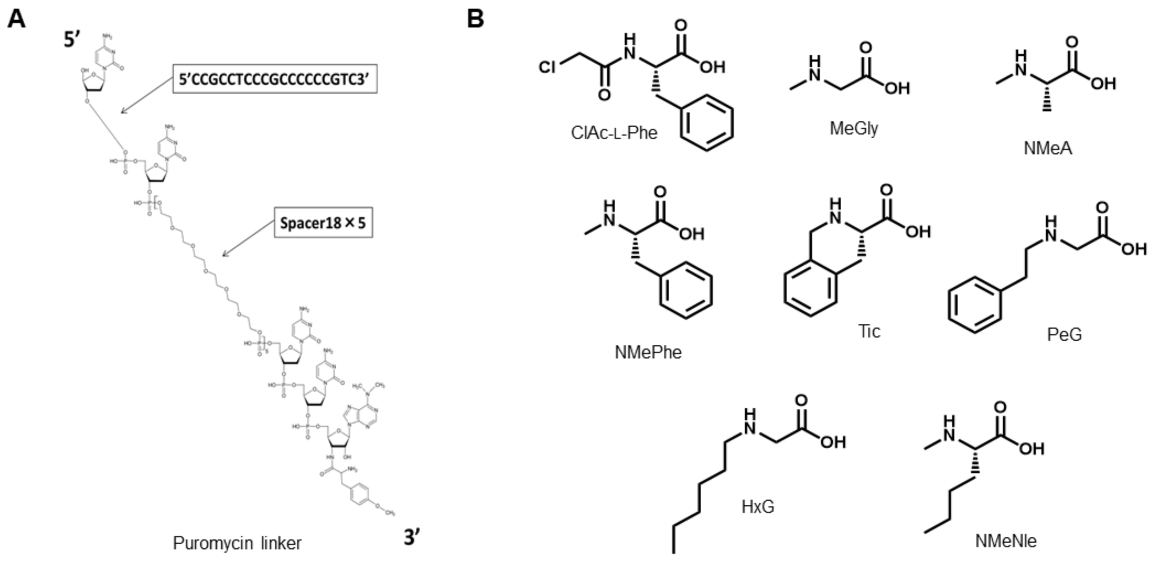

Fig. S1. Structures of (A) puromycin linker and (B) non-canonical amino acids used in the *in vitro* mRNA display selections.

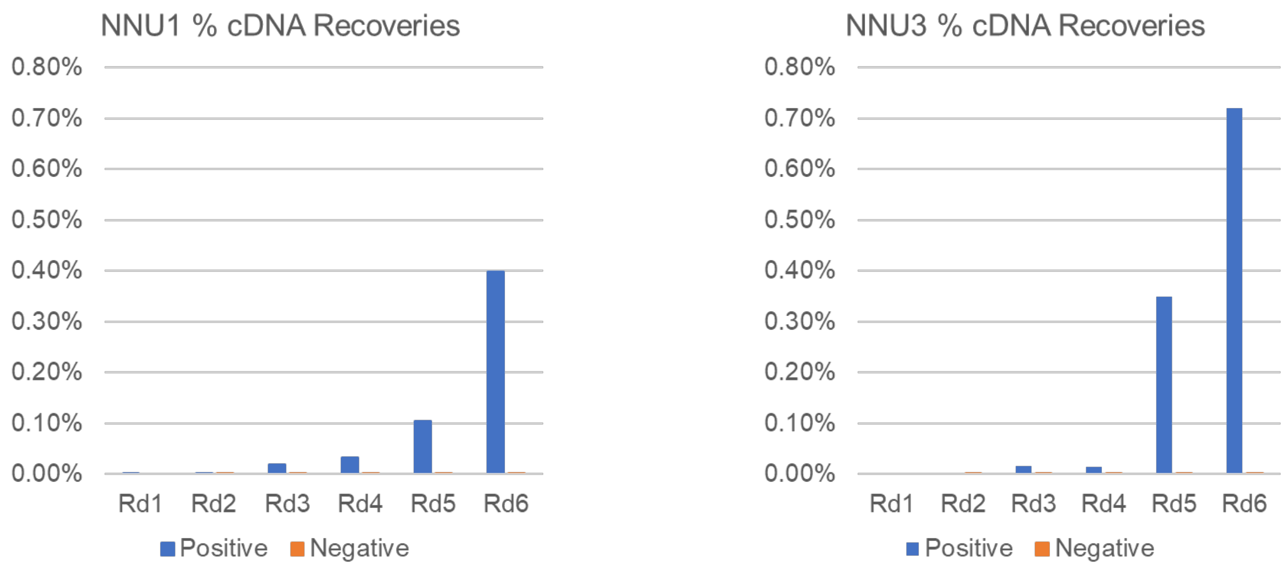

Fig. S2. Peptide enrichment was observed over six rounds of *in vitro* selection. The progress of the two *in vitro* selections was assessed using qPCR-based quantitation of the cDNA recovered after each round of selection. The percentage of the remaining cDNA sequences recovered by both positive selection (i.e., peptide-mRNA-cDNA pulled down by BamA) and negative selection (i.e., peptide-mRNA-cDNA pulled down by target-free beads) after each round is shown. The graphs show the results of a single experiment.

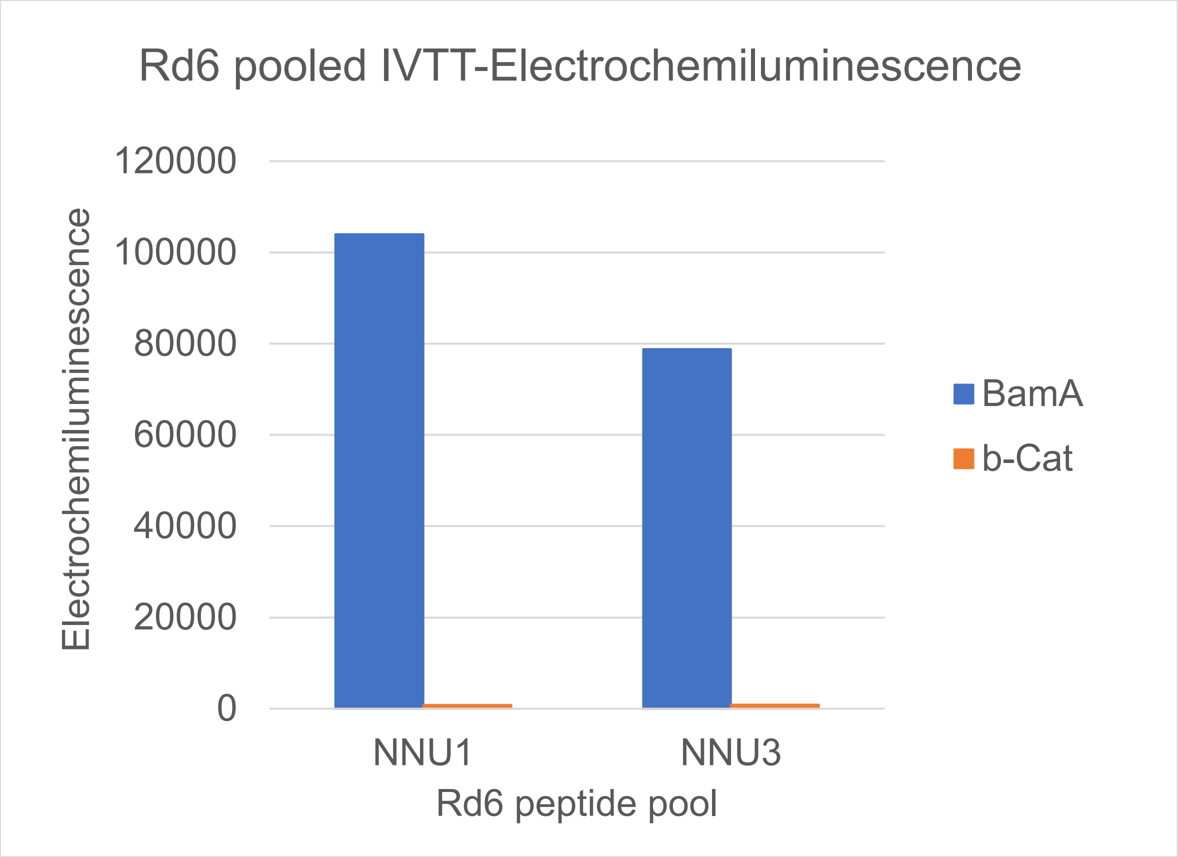

Fig. S3. The pooled macrocyclic peptides recovered after six rounds of *in vitro* selection show mRNA-independent binding to BamA. The post-round 6 peptide pools were modified to attach a C-terminal FLAG tag to each peptide so that it could be detected using an anti-FLAG tag primary antibody. An *in vitro* transcription-translation (IVTT)-coupled electrochemiluminescence assay was performed to assess BamA-specific peptide binding in the absence of the mRNA tag. Human β-catenin (b-Cat) was used as a negative control. The graphs show the results of a single experiment.

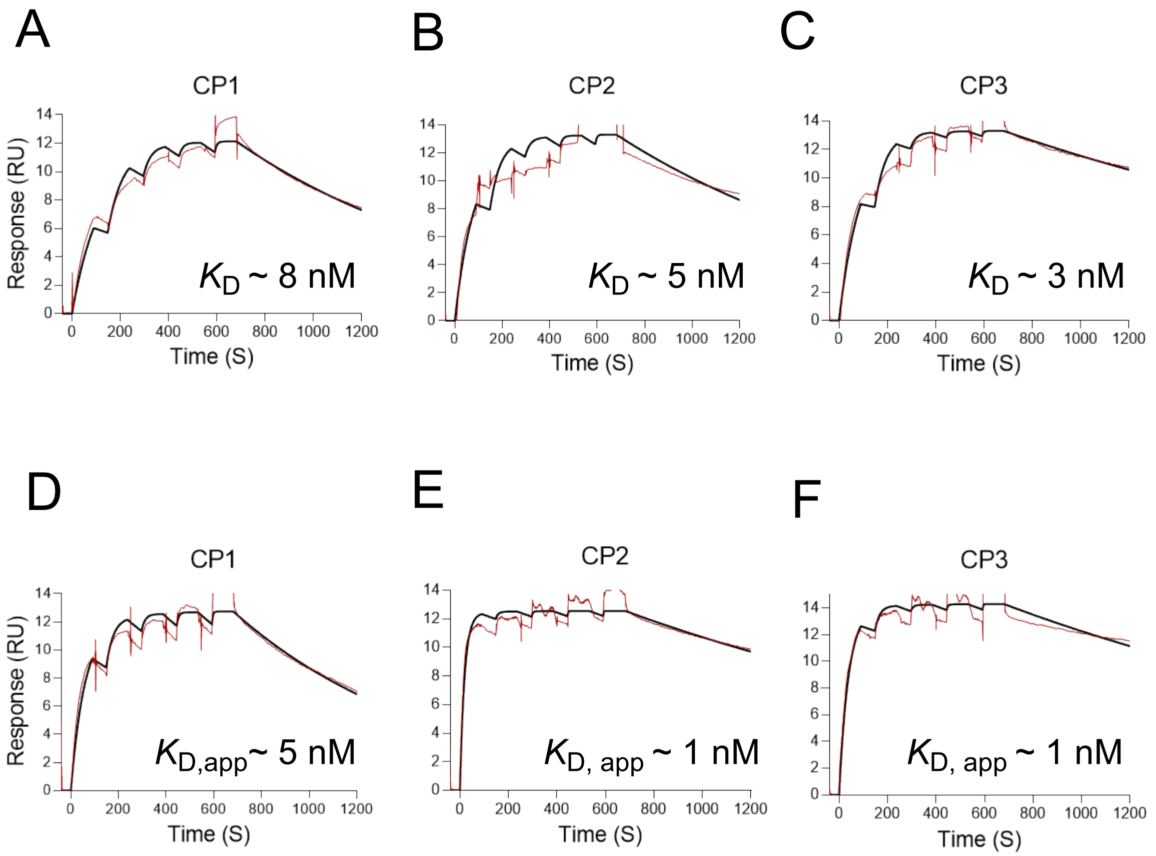

Fig. S4. Darobactin does not compete with mRNA display peptides. Peptide binding to the darobactin binding site in *E. coli* BamA was assessed using SPR. Affinity (K_D) for BamA was measured in the absence (A-C) or presence (D-F) of a saturating concentration of darobactin (i.e., BamA was pre-equilibrated with 2 μM darobactin as described in Methods). Because the presence of darobactin did not cause a significant shift in the apparent affinity ($K_{D,app}$) of the peptides for the BamA β-barrel it is very likely that the peptides and darobactin do not bind to the same site. The graphs show the results of a single experiment. Source data are provided as a Source Data file.

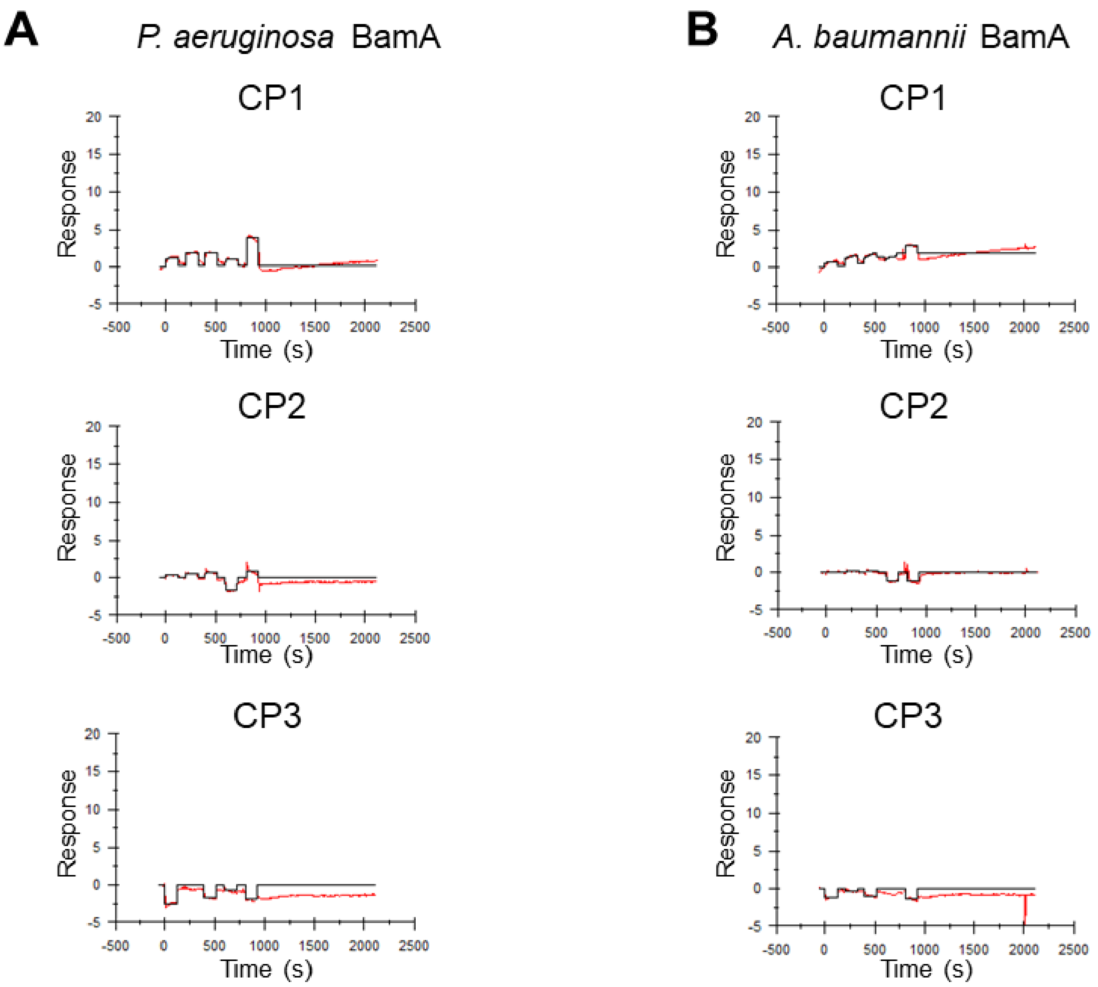

Fig. S5. Cyclic peptides isolated by mRNA display do not bind to the *P. aeruginosa* or *A. baumannii* BamA β-barrel. The interaction between the indicated peptide and the N-terminally biotinylated β-barrel of (A) *P. aeruginosa* or (B) *A. baumannii* BamA was analyzed by SPR. The sensorgrams show that none of the peptides bind to either protein. The graphs show the results of a single experiment.

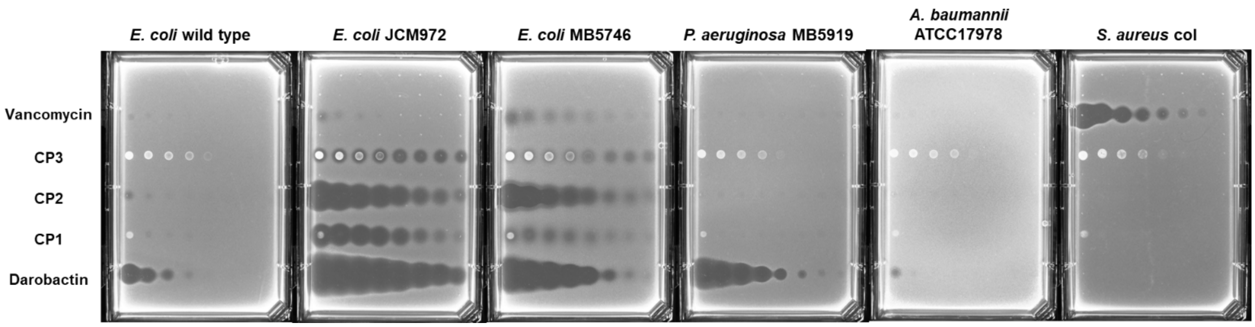

Fig. S6. Peptide activity against various bacterial strains. The agar plates above show the activity of CP1, CP2, CP3, darobactin A and vancomycin against various strains of *E. coli*, *Acinetobacter baumannii* ATCC17987, *Pseudomonas aeruginosa* MB5919, and *Staphylococcus aureus* Col. Darobactin showed activity against all Gram-negative strains, while CP1, CP2, and CP3 were only active against the two weakened *E. coli* strains JCM972 (*bamA101*) and MB5746 (*tolC*::Tn10 *lpxC (envA1)*). Compounds were diluted two-fold starting at a concentration of 1000 µM (vancomycin, darobactin) or 5000 µM (CP1, CP2, CP3), and 3 µL compound was used per spot.

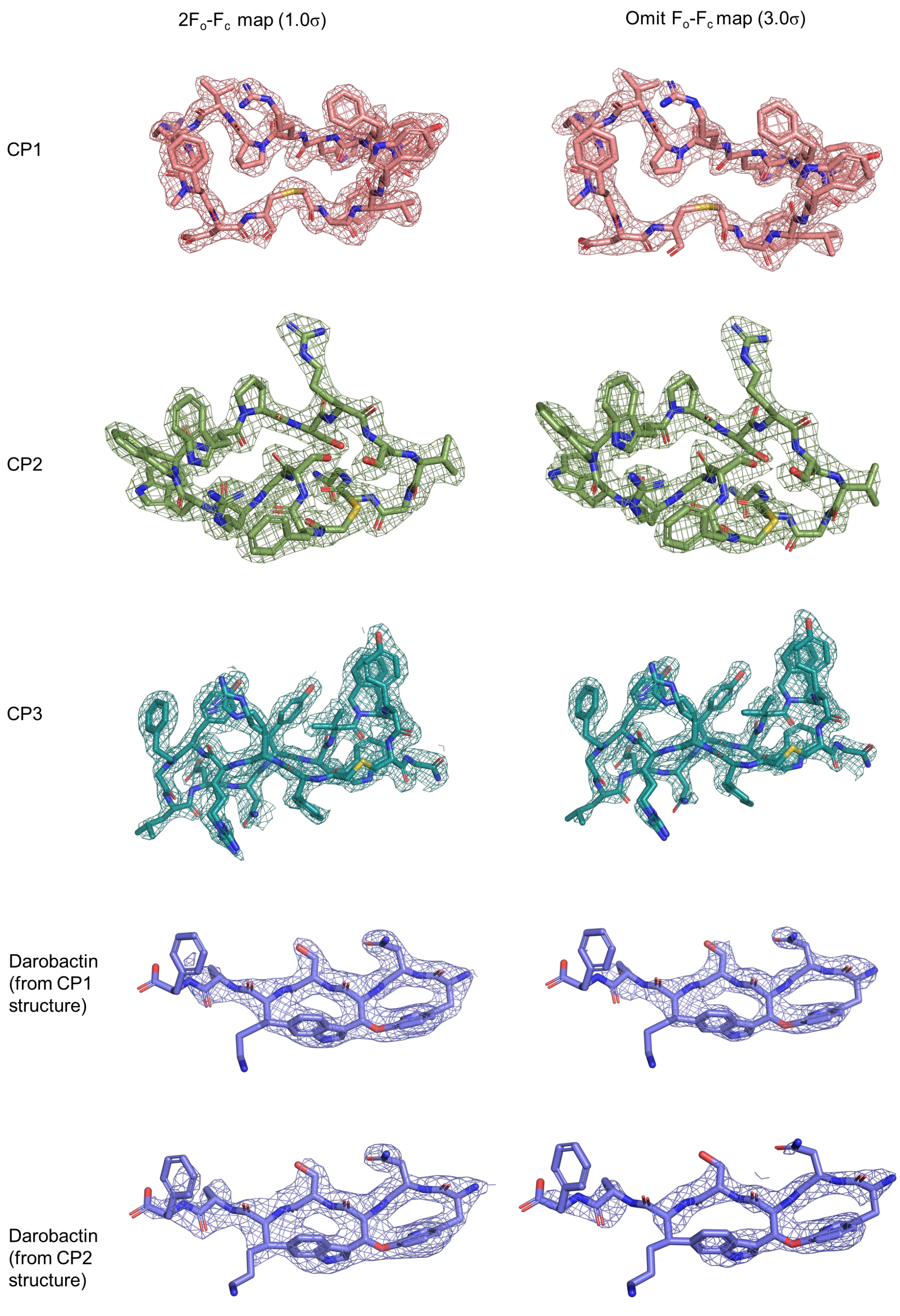

Fig. S7. 2Fo-Fc and omit maps for each compound described in the novel structures. The 2Fo-Fc maps that are shown are directly from refinement. Omit Fo-Fc maps were generated using the Refine method in the phenix.composite_omit_map tool[1–3] and selecting either the peptide or darobactin for omission. The 2Fo-Fc and Fo-Fc omit maps were generated in PyMOL at 1.0σ and 3.0σ, respectively.

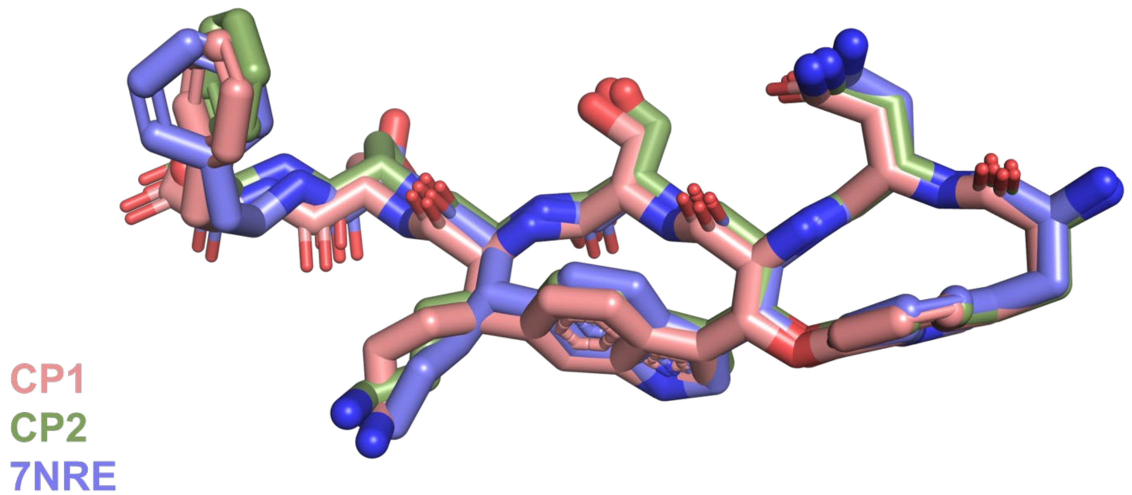

Fig. S8. An overlay of darobactin from extant and novel structures demonstrates that the binding modes are similar. To compare the binding mode and orientation of darobactin across crystal structures, the BamA β-barrels from indicated darobactin complex structures were aligned using the PyMOL align command. The resultant overlay of darobactin at the BamA lateral gate in each structure is shown above (without the BamA β-barrel for clarity).

A

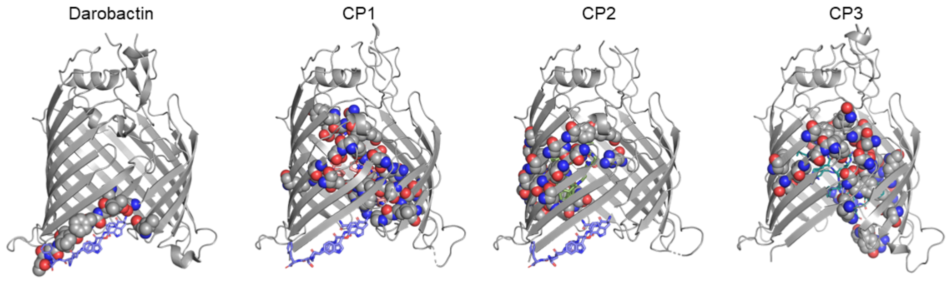

B

```
423-472
CP3:        TGSFNFGIGY  GTESGVSFQA  GVQQDNWLGT  GYAVGINGTK  NDYQTYAELS
CP1:        TGSFNFGIGY  GTESGVSFQA  GVQQDNWLGT  GYAVGINGTK  NDYQTYAELS
CP2:        TGSFNFGIGY  GTESGVSFQA  GVQQDNWLGT  GYAVGINGTK  NDYQTYAELS
Darobactin: TGSFNFGIGY  GTESGVSFQA  GVQQDNWLGT  GYAVGINGTK  NDYQTYAELS

473-522
CP3:        VTNPYFTVDG  VSLGGRLFYN  DFQADDADLS  DYTNKSYGTD  VTLGFPINEY
CP1:        VTNPYFTVDG  VSLGGRLFYN  DFQADDADLS  DYTNKSYGTD  VTLGFPINEY
CP2:        VTNPYFTVDG  VSLGGRLFYN  DFQADDADLS  DYTNKSYGTD  VTLGFPINEY
Darobactin: VTNPYFTVDG  VSLGGRLFYN  DFQADDADLS  DYTNKSYGTD  VTLGFPINEY

523-572
CP3:        NSLRAGLGYV  HNSLSNMQPQ  VAMWRYLYSM  GEHPSTSDQD  NSFKTDDFTF
CP1:        NSLRAGLGYV  HNSLSNMQPQ  VAMWRYLYSM  GEHPSTSDQD  NSFKTDDFTF
CP2:        NSLRAGLGYV  HNSLSNMQPQ  VAMWRYLYSM  GEHPSTSDQD  NSFKTDDFTF
Darobactin: NSLRAGLGYV  HNSLSNMQPQ  VAMWRYLYSM  GEHPSTSDQD  NSFKTDDFTF

573-622
CP3:        NYGWTYNKLD  RGYFPTDGSR  VNLTGKVTIP  GSDNEYYKVT  LDTATYVPID
CP1:        NYGWTYNKLD  RGYFPTDGSR  VNLTGKVTIP  GSDNEYYKVT  LDTATYVPID
CP2:        NYGWTYNKLD  RGYFPTDGSR  VNLTGKVTIP  GSDNEYYKVT  LDTATYVPID
Darobactin: NYGWTYNKLD  RGYFPTDGSR  VNLTGKVTIP  GSDNEYYKVT  LDTATYVPID

623-672
CP3:        DDHKWVVLGR  TRWGYGDGLG  GKEMPFYENF  YAGGSSTVRG  FQSNTIGPKA
CP1:        DDHKWVVLGR  TRWGYGDGLG  GKEMPFYENF  YAGGSSTVRG  FQSNTIGPKA
CP2:        DDHKWVVLGR  TRWGYGDGLG  GKEMPFYENF  YAGGSSTVRG  FQSNTIGPKA
Darobactin: DDHKWVVLGR  TRWGYGDGLG  GKEMPFYENF  YAGGSSTVRG  FQSNTIGPKA

673-722
CP3:        VYFPHQASNY  DPDYDYECAT  QDGAKDLCKS  DDAVGGNAMA  VASLEFITPT
CP1:        VYFPHYEC--  ----------  --AAKDLCKS  DDAVGGNAMA  VASLEFITPT
CP2:        VYFP------  ----------  ------LCKS  DDAVGGNAMA  VASLEFITPT
Darobactin: VYFPHQ----  ----------  -----DLSKS  DDAVGGNAMA  VASLEFITPT

723-772
CP3:        PFISDKYANS  VRTSFFWDMG  TVWDTNWDSS  QYSGYPDYSD  PSNIRMSAGI
CP1:        PFISD--ANS  VRTSFFWDMG  TVWDTNWDSS  QYSGYPDYSD  PSNIRMSAGI
CP2:        PFIS---ANS  VRTSFFWDMG  TVWDTNWDSS  QYSGYPDYSD  PSNIRMSAGI
Darobactin: PFISDKYANS  VRTSFFWDMG  TVWDTNWDSS  QYSGYPDYSD  PSNIRMSAGI

773-810
CP3:        ALQWMSPLGP  LVFSYAQPFK  KYDGDKAEQF  QFNIGKTW
CP1:        ALQWMSPLGP  LVFSYAQPFK  KYDGDKAEQF  QFNIGKTW
CP2:        ALQWMSPLGP  LVFSYAQPFK  KYDGDKAEQF  QFNIG---
Darobactin: ALQWMSPLGP  LVFSYAQPFK  KYDGDKAEQF  QFNIGKTW
```

- ligand interacts with one or more polar atoms – hydrogen bond, ionic, cation-dipole, cation-pi, dipolar, halogen, donor-pi
- ligand interacts with one or more apolar atoms - pi-pi, Vanderwaals
- ligand interacts with both polar and apolar atoms
- ligand has both favourable and unfavourable interactions
- ligand is nearby but forms no favourable contacts
- ligand has one or more unfavourable interactions

Fig. S9. Summary of BamA residues critical for cyclic peptide binding. (A) The cyclic peptides isolated by mRNA display bind to different regions of the *E. coli* BamA β-barrel than darobactin. Binding residues are shown as spheres. (B) A residue interaction diagram for each cyclic peptide and darobactin based on the crystal structures is shown.

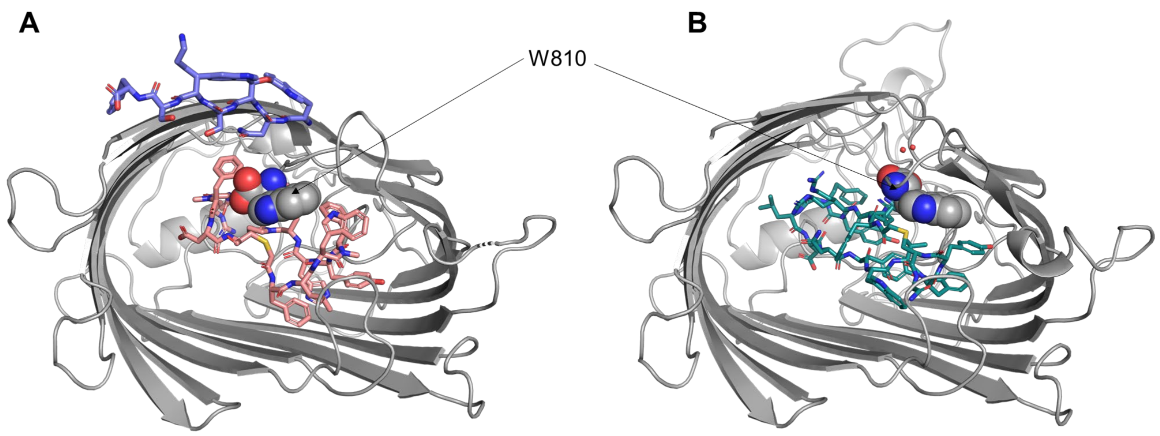

Fig. S10. Gate-binding peptides trap the C-terminal residue of BamA in the β-barrel lumen. The *E. coli* BamA residue W810 in the presence of (A) CP1 (pink) and (B) CP3 (teal) is shown as spheres. Darobactin is shown in blue at the top of A.

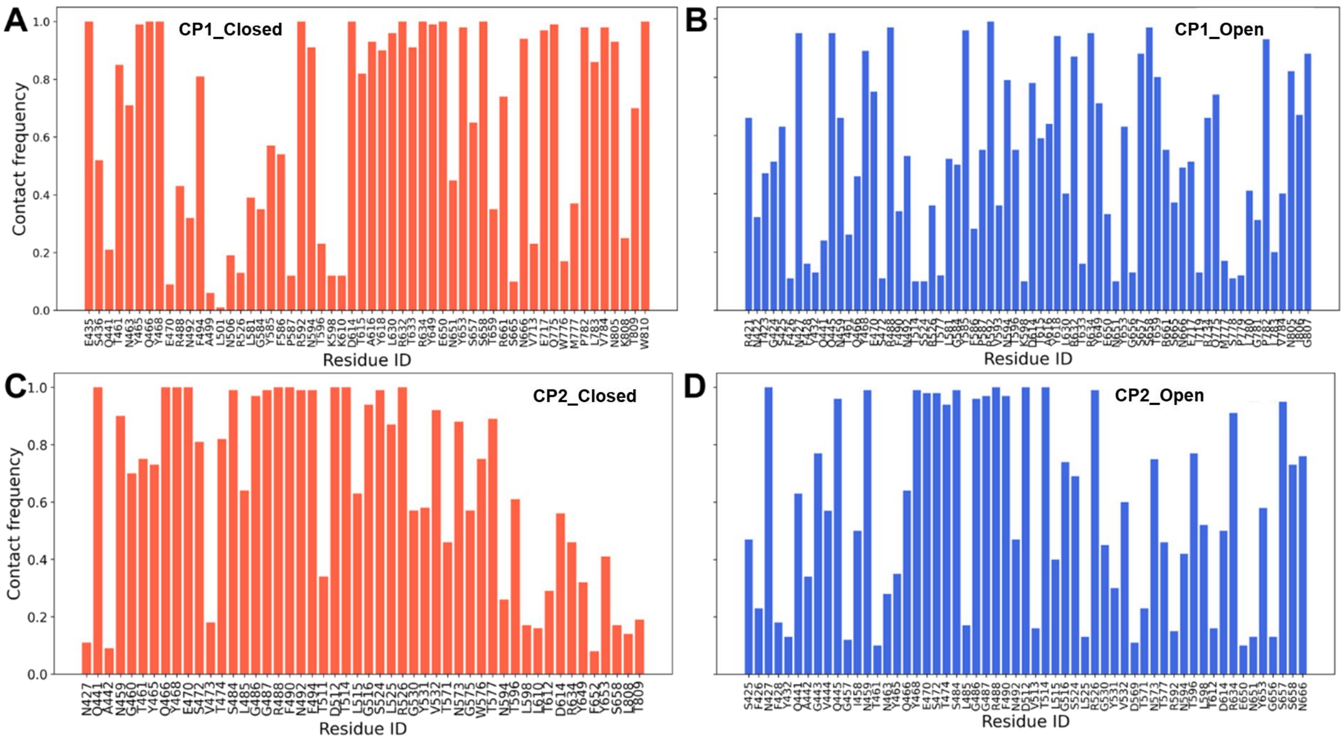

Fig. S11. Identification of BamA residues that exhibit a high contact frequency with the cyclic peptides in the MD simulations across three replicas. The contact frequency of residues in simulations of (A) CP1 in the closed BamA state, (B) CP1 in the open BamA state, (C) CP2 in the closed BamA state and (D) CP2 in the open BamA state are shown. The contact frequency represents the fraction of the total simulation time during which a given residue is within 4 Å of the peptide.

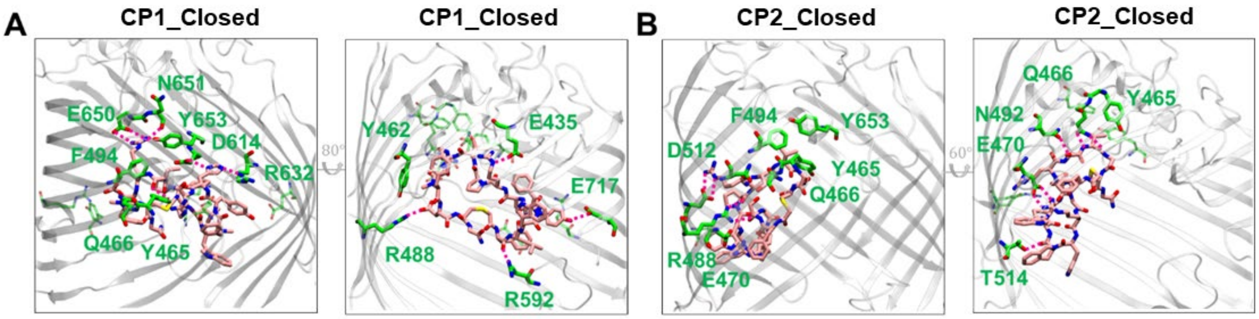

Fig. S12. Key residues involved in interactions with (A) CP1 and (B) CP2 in MD simulations.

A

Legend:

Fully conserved	Properties strongly conserved	Properties weakly conserved	Non-conserved

```
Ec_MG1655              KVKERNTGSFNFGIGYGTESGVSFQAGVQQDNWLGTGYAVGINGTKNDYQTYAELSVTNP 476
Kp_strain342           KVKERNTGSFNFGIGYGTESGVSFQAGVQQDNWLGTGYAVGINGTKNDYQTYTELSVTNP 476
Enterobacter_sp_638    KVKERNTGSFNFGVGYGTESGVSFQVGVQQDNWLGTGYSVGINGTKNDYQTYSEFSVTNP 476
C_freundii             KVKERNTGSFNFGVGYGTESGVSFQAGVQQDNWLGTGYSVGINGTKNDYQTYTELSVTNP 476
S_typhimurium_LT2      KVKERNTGSFNFGIGYGTESGVSFQAGVQQDNWLGTGYSVGINGTKNDYQTYSELSVTNP 476
Ab_ATCC19606           NVEEQHSGTTTLAVGYSQSGGITFQAGLSQTNFMGTGNRVAIDLSRSETQDYYNLSVTDP 480
PA_PAO1                SVEEQPSGSITASVGFAQSAGLILGGSISQNNFLGTGNKVSIGLTRSEYQTRYNFGFVDP 475
                       .*:*: :*: . .:*:. ..*: :  .:.* *::***  *.*. ::.: *   ::...:*

Ec_MG1655              YFTVDGVSLGGRLFYNDFQAD--DADLSDYTNKSYGTDVTLGFPINEYNSLRAGLGYVHN 534
Kp_strain342           YFTVDGVSLGGRIFYNDFDAN--DADLSDYTNKSYGTDVTLGFPVNEYNTLRAGVGYVHN 534
Enterobacter_sp_638    YFTVDGVSLGGRIFYNDFKAD--DADLSSYTNKSYGLDGTLGFPINEYNTLRAGLGYVHN 534
C_freundii             YFTVDGVSLGGRVFYNDFEAD--DADLSDYTNKSYGTDVTLGFPINEYNTLRAGLGYVHN 534
S_typhimurium_LT2      YFTVDGVSLGGRIFYNDFQAD--DADLSDYTNKSYGTDVTLGFPINEYNTLRAGLGYVHN 534
Ab_ATCC19606           YFTIDGVSRGYNVYYRKTKLND-DYNVNNYVTDSFGGSLSFGYPIDENQSLSASVGVDNT 539
PA_PAO1                YWTVDGVSLGYNAFYRKTDYDELDVDVASYSVNSLGAGMSIGYPISETSRLTYGLSVQRD 535
                       *:*:**** *  . :*.. . :  * :: .*  .* * . ::*:*:.* . *  .:.  .

Ec_MG1655              SLSNMQPQ-VAMWRYLYSMGEHPST--------------------------SDQDNSFK- 566
Kp_strain342           SLSNMQPQ-VAMWRYLNSMGQYPDN--------------------------TNDRNSFS- 566
Enterobacter_sp_638    DLSNMQPQ-VAMWRYLDSIGQAASK--------------------------SSDNNGFA- 566
C_freundii             SLSNMDPQ-VAMQRYLVSMGEDPDA------------------------------DSFK- 562
S_typhimurium_LT2      KLSNMQPQ-IAMDRYLESMGQSAD------------------------------TSSFA- 562
Ab_ATCC19606           KVTTGPYVSTYVRDYLLANGGKATSKGTYCPTDANGDSQYDTEKGECKVPEETYDNAFEG 599
PA_PAO1                QIDTGRYTVDEIYDFLDKE---------------------------------------GD 556
                       .: .        :  :*

Ec_MG1655              -TDDFTFNYGWTYNKLDRGYFPTDGSRVNLTGKVTIPGSDNEYYKVTLDTATYVPIDDDH 625
Kp_strain342           -ANDFTFNYGWTYNKLDRGFFPTEGSRVNLNGKVTIPGSDNEYYKATLDTATYVPIDNDH 625
Enterobacter_sp_638    -ADDFTFNYGWTYNRLDRGFFPTEGSRVNLNGKVTVPGSDNEFYKLTLDTASYFPIDDDH 625
C_freundii             -SDDFTFNYGWTYNKLDRGYFPTEGTRVNLNGKVTVPGSDNEYYKATLDTATYVPIDDDH 621
S_typhimurium_LT2      -ADDFTFNYGWTYNKLDRGYFPTDGSRVNLTGKVTIPGSDNEYYKVSLDTATYVPIDNDH 621
Ab_ATCC19606           EFFTYNLNLGWSYNTLNRPIFPTSGMSHRVGLEIGLPGSDVDYQKVTYDTQAFFPIGST- 658
PA_PAO1                NFTNFKASIGWSESTLNKGVLATRGHSQSLTLETTLPGSDLSFYKIDYRGQVFAPLTDN- 615
                           :. . **: . *::  : * *    :  :  :**** .: *       : *: .

Ec_MG1655              KWVVLGRTRWGYGDGLG-GKEMPFYENFYAGGSSTVRGFQSNTIGPKAVYFPHQASNYDP 684
Kp_strain342           QWVVLGRTRFGYGDGIG-GKEMPFYENFYAGGSSTVRGFQSNTIGPKAVYFPSSSRHD-G 683
Enterobacter_sp_638    KWVVLGRTRWGYGDGLG-GKELPFYENFYAGGSSTVRGFQSNNIGPKAVYYGGN------ 678
C_freundii             KWVVLGRTKWGYGDGLS-GKEMPFYENFYAGGSSTVRGFQSNTIGPKAVYLPANRYNDDD 680
S_typhimurium_LT2      KWVVLGRTRWGYGDGLG-GKEMPFYENFYAGGSSTVRGFQSNTIGPKAVYKNGAHTSWDD 680
Ab_ATCC19606           GFVLRGYGKLGY------GNDLPFYKNFYAGGYGSVRGYDNSTLGPKYPSVNLQ------ 706
PA_PAO1                -YTMRFHTELGYGDGYGSTERLPFYENYYAGGFNSVRGFKDSTLGPRSTPSVARNPDGTP 674
                        :.:     . **        : :***:*:****  .:***:....:**:

Ec_MG1655              DYDYECATQDGAKDLCKSDDAVGGNAMAVASLEFITPTPFISDKYANSVRTSFFWDMGTV 744
Kp_strain342           DSGYTNDCKSTESAPCKSDDAVGGNAMAVASLELITPTPFISDKYANSVRTSVFWDMGTV 743
Enterobacter_sp_638    --DKDNCNKTSSSEVCSSDDAVGGNAMGVASLEIITPTPFISDKYANSVRTSFFWDAGTV 736
C_freundii             DYDNECDTTN--TSPCKSDDAVGGNAMAVASLEFITPTPFISDKYANSVRTSFFWDMGTV 738
S_typhimurium_LT2      NDDYEDCTQE---SGCKSDDAVGGNAMAVASLEFITPTPFISEKYANSVRTSFFWDMGTV 737
Ab_ATCC19606           ----------ETKQNDSSPEEVGGNALVQFGTELVLPMPFKGD-WTRQVRPVLFAEGGQV 755
PA_PAO1                MKNQGPDSKGRYTDPDQDPEAFGGNILITGGAELLFPLPFVKD--QRQLRTVLFWDVGST 732
                                        .. : .*** :   . *:: * **  :    ..:*  .* : * .

Ec_MG1655              WDTNWDSSQ--------------------YSGYPDYSDPSNIRMSAGIALQWMSPLGPLV 784
Kp_strain342           WDTHWDSN---------------------AYGGYPDYSDPSNIRMSAGIAVQWMSPLGPLV 783
Enterobacter_sp_638    WDTNWENTAQM------------------KAAGVPDYSDPSNIRMSAGIALQWMSPLGPLV 779
C_freundii             WDTNWDSAK--------------------YSGYPDYSDPSNIRMSAGIALQWMSPLGPLV 778
S_typhimurium_LT2      WDTNWDPSS--------------------APSDVPDYSDPGNIRMSAGIALQWMSPLGPLV 778
Ab_ATCC19606           FDTKCNIDNSVYGNKGMKINGQTITDVRKYCEDNYGFDLGNLRYSVGVGVTWITMIGPLS 815
PA_PAO1                FDTDCPTKTTT---------------------NCDGIKTDNLASSVGVGLTWITALGPLS 771
                       :**.                                  . .*:  *.*:.: *:: :***
```

```
Ec_MG1655              FSYAQPFKKYDGDKAEQFQFNIGKTW      810
Kp_strain342           FSYAQPFKKYDGDKAEQFQFNIGKTW      809
Enterobacter_sp_638    FSYAQPFKKYDGDKAEQFQFNIGKTW      805
C_freundii             FSYAQPFKKYDGDKAEQFQFNIGKTW      804
S_typhimurium_LT2      FSYAQPFKKYDGDKAEQFQFNIGKTW      804
Ab_ATCC19606           LSYAFPLNDKPGDETKEIQFEIGRTF      841
PA_PAO1                FSLATPIKKPDNAETQVFQFSLGQTF      797
                       :* * *::.  . ::: :**.:*:*:
```

B

	Binding residues in *E. coli* BamA that are:			
Peptide	Fully conserved	Properties strongly conserved	Properties weakly conserved	Non-conserved
CP1	Q466, Y649, N651, Y653, E717, P782, L783, T809	T461, E650, W810	E435, R488, R634, S658, N805	Y465, Y468, R592, D614, A616, R632, Q775, V784
CP2	Q466, S484, G486, L525	E470, F490, T514	N459, R488, N492, D512	Y468, G487, F494, S524, R526
CP3	Q466, K610, Y653, R661	E470, F490, R583, K808, W810	E435, R634, S658, N666	Y465, Y468, F494, G584, Y585, P587, R592, R632

Fig. S13. BamA residues that are important for peptide binding are highly conserved among Enterobacteriaceae but not other Gram-negative bacteria. (A) Multiple sequence alignment of the BamA β-barrel from Enterobacteriaceae [*E. coli* (Ec_MG1655), *Klebsiella pneumoniae* (Kp_strain342)*, Enterobacter sp.* (strain 638), *Citrobacter freundii* (Uniprot A0AAJ4HCI4), *Salmonella typhimurium* (strain LT2)], *A. baumannii* (Ab_ATCC19606), and *P. aeruginosa* (PA_PAO1). Residues that interact with CP1, CP2, or CP3 are highlighted in yellow (*, fully conserved in organisms shown here), pink (:, overall properties strongly conserved in organisms shown here), green (., overall properties weakly conserved in organisms shown here) or blue (non-conserved). All of the peptide binding residues are identical in the Enterobacteriaceae shown here except S524 (which is a threonine in some species) and R634 (which is a lysine in *C. freundii*). The alignment was created with CLUSTAL O(1.2.4). (B) Table showing conservation of residues involved in the binding of each peptide.

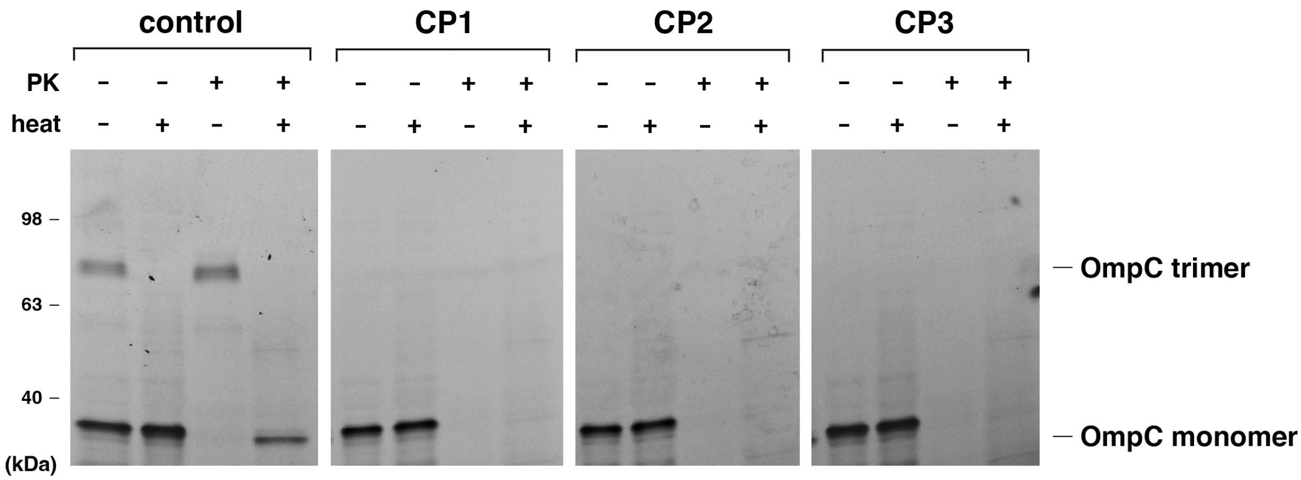

Fig. S14. Cyclic peptides CP1, CP2, and CP3 inhibit the membrane integration of OmpC monomers *in vitro*. PURExpress coupled transcription/translation reactions were performed as in Fig. 4 to examine the effect of the cyclic peptides (at a concentration of 200x K_D) on the assembly of OmpC except that half of each reaction was treated with 30 μg/ml PK on ice for 15 min. Half of each sample was then heated to 95° C while the other half was left unheated, and proteins were resolved by SDS-PAGE. Because no PK-resistant OmpC monomer was observed when a cyclic peptide was added to the reaction, the data indicate that the peptides block the integration of OmpC monomers into the BAM-POPC proteoliposomes. The gels show the results of a single experiment.

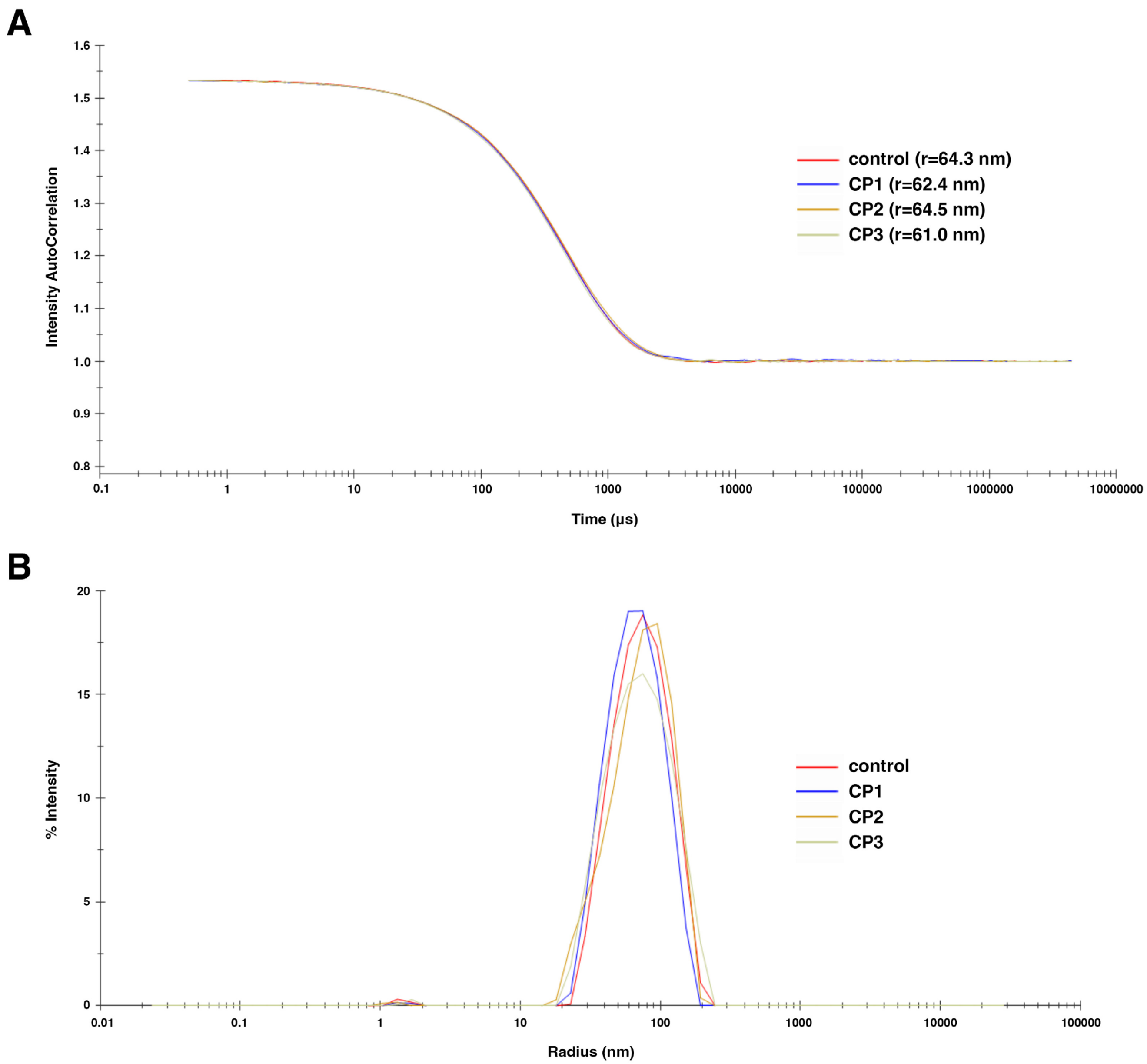

Fig. S15. The cyclic peptides do not affect the structure of BAM-POPC proteoliposomes. The indicated peptide (or buffer, control) was added at a concentration of 200x K_D to 0.5 μM BAM-POPC in 20 mM Tris pH 8.0, incubated at 30° C for 10 min, and placed on ice. The samples were then analyzed using dynamic light scattering (DLS). The results are plotted as an intensity autocorrelation curve (A) and as a particle size distribution curve (B). Source data are provided as a Source Data file.

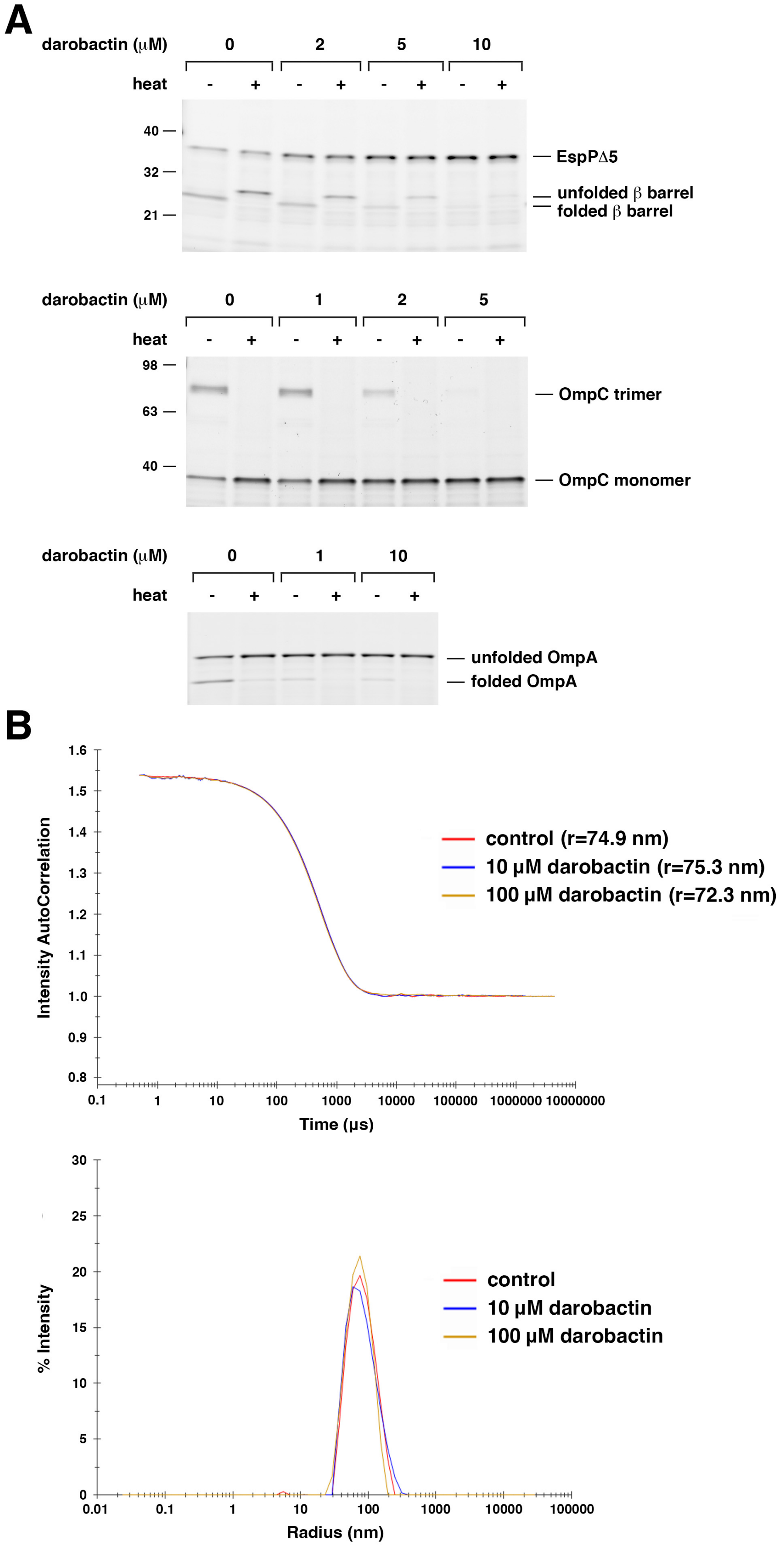
A
darobactin (μM)
0
2
5
10
heat
- + - + - + - +
40
32
21
EspPΔ5
unfolded β barrel
folded β barrel
darobactin (μM)
0
1
2
5
heat
- + - + - + - +
98
63
40
OmpC trimer
OmpC monomer
darobactin (μM)
0
1
10
heat
- + - + - +
unfolded OmpA
folded OmpA
B
Intensity AutoCorrelation
Time (μs)
control (r=74.9 nm)
10 μM darobactin (r=75.3 nm)
100 μM darobactin (r=72.3 nm)
% Intensity
Radius (nm)
control
10 μM darobactin
100 μM darobactin

Fig. S16. Darobactin inhibits the assembly of OMPs *in vitro*. (A) PURExpress coupled transcription/translation reactions were performed as in Fig. 4 to examine the effect of darobactin at the indicated concentrations on the assembly of EspPΔ5', OmpC and OmpA *in vitro*. The EspPΔ5' assembly assay was performed twice with similar results, and the OmpC and OmpA assembly assays were performed once. (B) Darobactin (or buffer, control) was added at the indicated concentration to 0.5 μM BAM-POPC in 20 mM Tris pH 8.0, incubated at 30° C for 10 min, and placed on ice. The samples were then analyzed using dynamic light scattering (DLS). The results are plotted as an intensity autocorrelation curve (top) and as a particle size distribution curve (bottom). Source data are provided as a Source Data file.

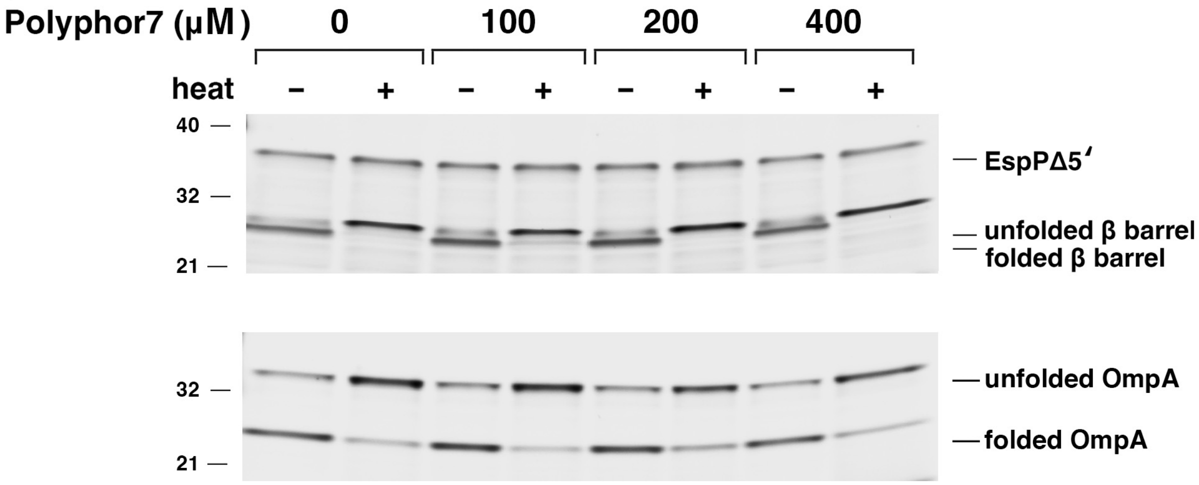

Fig. S17. Polyphor7 does not affect the assembly of EspPΔ5' or OmpA *in vitro* even at high concentrations. PURExpress coupled transcription/translation reactions were performed as in Fig. 4 to examine the effect of Polyphor7 (a compound that is similar to Polyphor3, which has a reported K_D of ~0.2 µM[4]) at the indicated concentrations on the assembly of EspPΔ5' *in vitro*. The gels show the results of a single experiment.

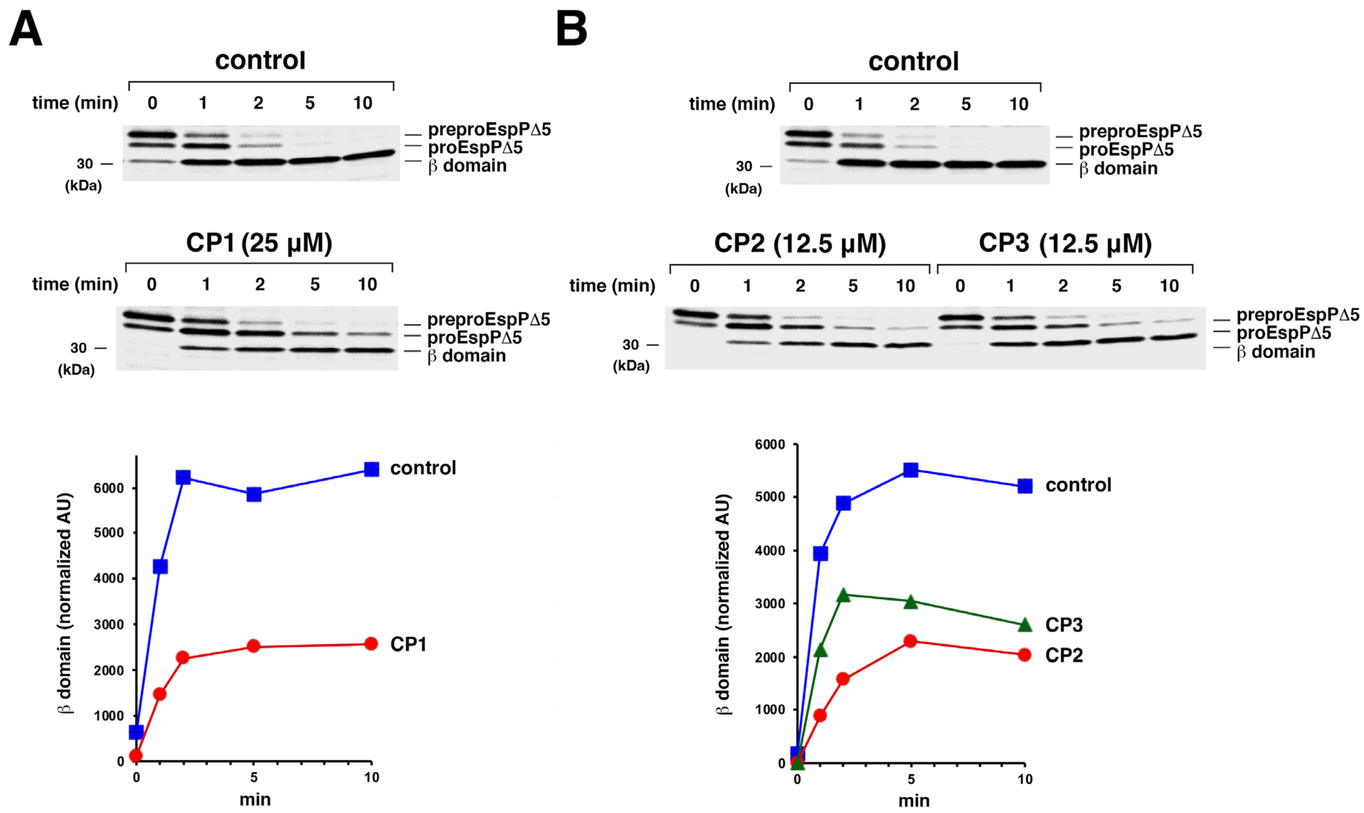

Fig. S18. Cyclic peptides only partially inhibit the assembly of a model OMP in a wild-type strain. The experiments described in Fig. 5 with (A) CP1 and (B) CP2 and CP3 were repeated except that strain AD202 was used and only the assembly of EspPΔ5' was evaluated. The experiments shown here were performed twice with similar results.

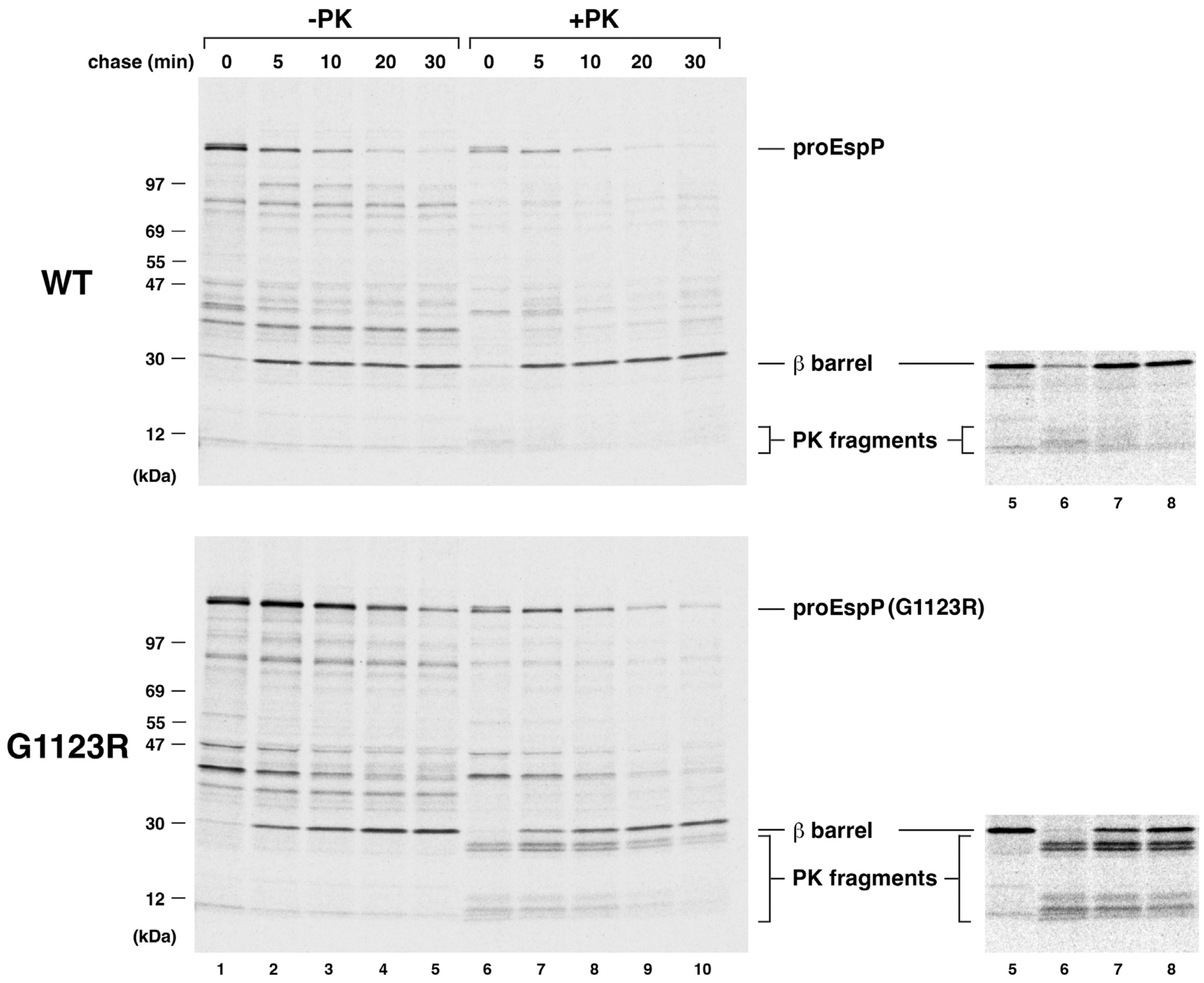

Fig. S19. The β-barrel of the EspP (G1123R) mutant is partially inserted into the OM after its β signal binds to BamA β1. The basic experimental protocol described in Fig. 6 was repeated using AD202 transformed with plasmid pRLS5 [P_{trc}-*espP*] or pJH224 [P_{trc}-*espP*(G1123R)], except that no compounds were added to the cultures either before or after the start of the chase, and 1 ml aliquots of radiolabeled cells were pipetted over ice at each timepoint. After cells were collected by centrifugation, resuspended in 33 mM Tris pH 8.0/40% sucrose and the OM was permeabilized, half of each sample was treated with PK while the other half was left untreated. Proteins were precipitated with TCA, and immunoprecipitations were conducted using an anti-EspP C-terminal peptide. The persistence of ~26-28 kDa and ~12-14 kDa C-terminal fragments of the EspP(G1123R) mutant (but not wild-type EspP) after PK treatment indicates that a large portion of the β-barrel was inserted into the OM after the β-signal bound to BamA β1 but before the protein was fully assembled. For clarity, the bottom part of lanes 5-8 was overexposed and shown on the right. This experiment was performed twice with similar results.

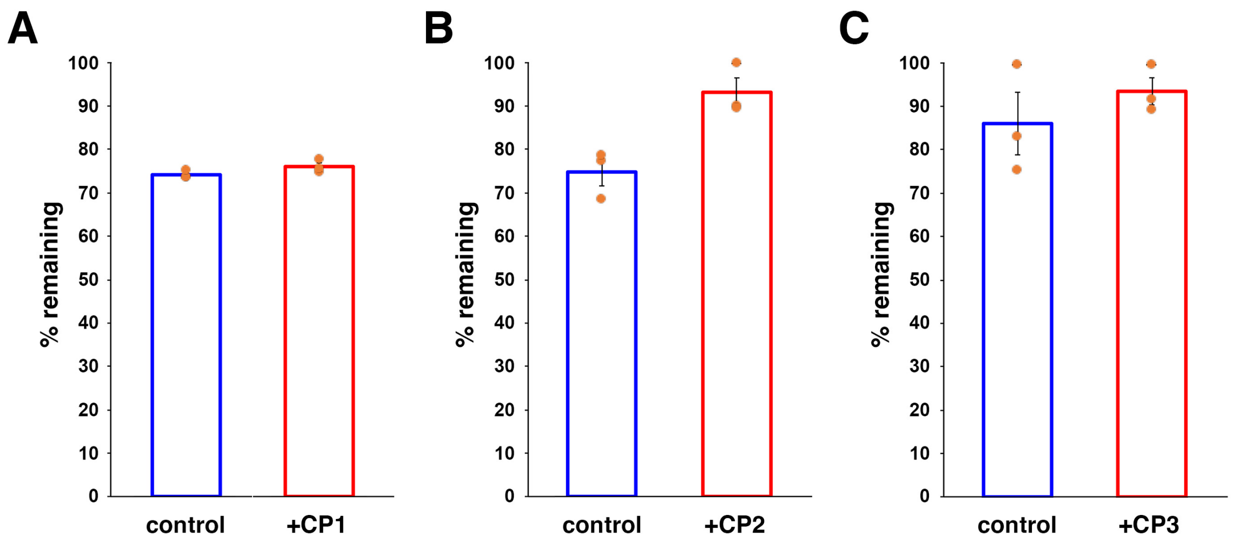

Fig. S20. Cyclic peptides do not reduce the stability of the EspP(G1123R) mutant. The stability of the EspP(G1123R) mutant in cells that were untreated (control) or treated 2 min after the start of the chase with peptide CP1 (A), CP2 (B), or CP3 (C) in the experiments shown in Fig. 6 was determined by calculating the percent of the radiolabeled protein that was observed at the 5 min timepoint that remained at the 30 min timepoint. The maximum value was 100%. Data are presented as mean values and error bars represent the standard error of the mean (SEM). Source data are provided as a Source Data file.

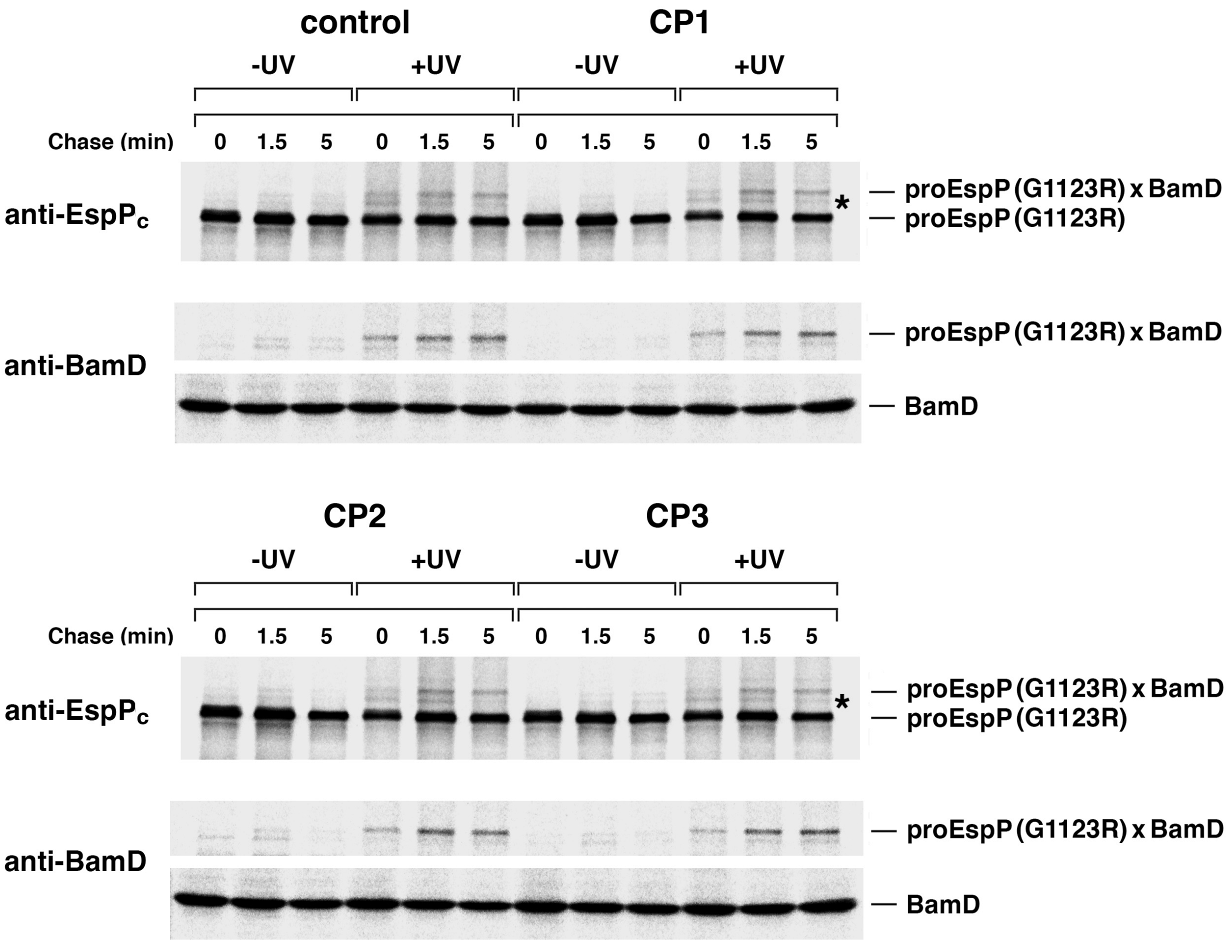

Fig. S21. The cyclic peptides do not promote the dissociation of EspP(G1123R) from BAM. AD202 transformed with a derivative of pRI22 [P_{lac}-*espP*(F1214am)] that contains the EspP(G1123R) mutation and pDULE were grown to mid-log phase, 1 mM Bpa was added, and cells were subjected to pulse-chase radiolabeling 30 min later. The indicated peptide (2x MIC) was added to cultures 2 min after the start of the chase. At each timepoint cells were either untreated and pipetted over ice or UV-irradiated. Immunoprecipitations with antisera directed against an EspP C-terminal peptide and BamD were subsequently performed and proteins were resolved by SDS-PAGE. It was previously shown that Bpa introduced at EspP residue 1214 by amber suppression can be crosslinked to BamD2. Based on previous studies, the band labelled with an asterisk is likely proEspP(G1123R) crosslinked to Skp[5,6]. For clarity, the region of the gels that were run following immunoprecipitations with the anti-BamD antiserum between the proEspP(G1123R)-BamD crosslinking product and BamD is not shown. The gels show the results of a single experiment.

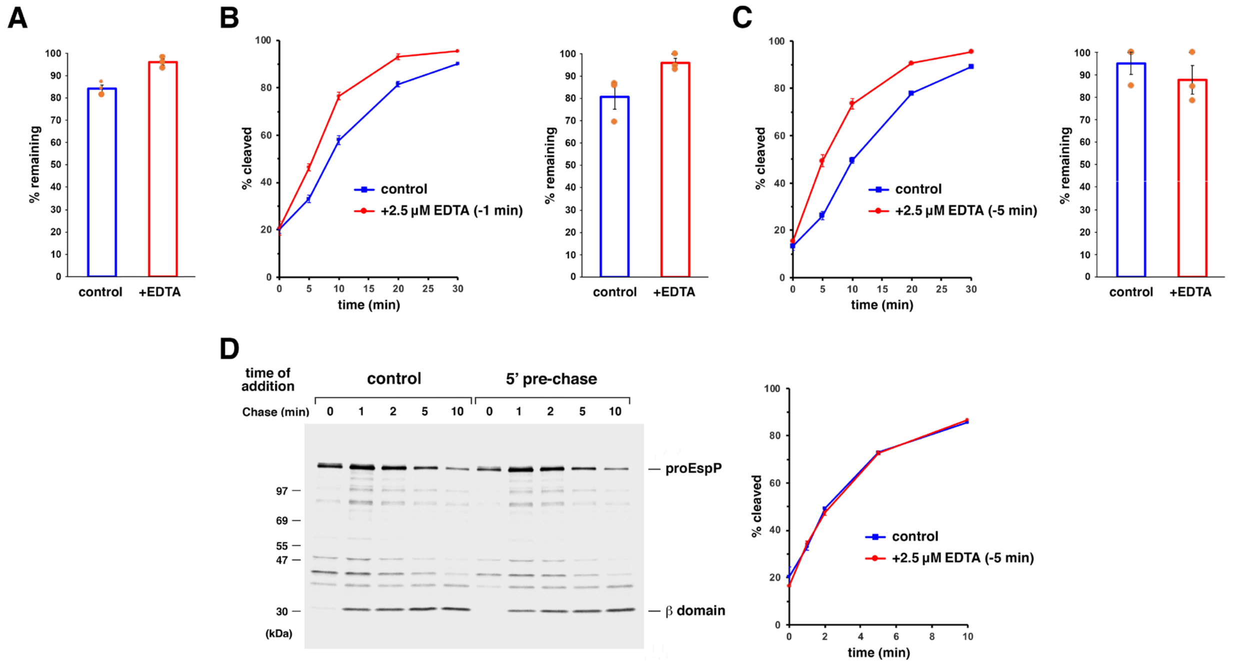

Fig. S22. EDTA accelerates the assembly of EspP(G1123R) but not wild-type EspP if added prior to synthesis. (A) The percent of the radiolabeled protein that remained at the 30 min time point in the three independent experiments described in Fig. 6C was calculated as described in the legend to Fig. S20. (B-C) The experiments described in Fig. 6C were repeated except that the EDTA was added at 1 min (B) or 5 min (C) before the start of the chase. The assembly of EspP(G1123R) was quantitated on the left by calculating the percent of the protein that underwent autocatalytic cleavage in cells that had been treated with EDTA or untreated in three independent experiments, and the percent of the radiolabeled protein that remained at the 30 min time point is shown on the right. (D) The experiments shown in Fig. 6C were repeated except that AD202 transformed with pRLS5 (P_{trc}-*espP*) were either untreated or treated with 2.5 mM EDTA 5 min prior to the start of the chase. A gel from a representative experiment is shown on the left. The assembly of EspP in three biologically independent experiments was quantitated on the right by calculating the percent of protein that underwent autocatalytic cleavage. In all experiments data are presented and mean values and error bars represent the SEM. Source data are provided as a Source Data file.

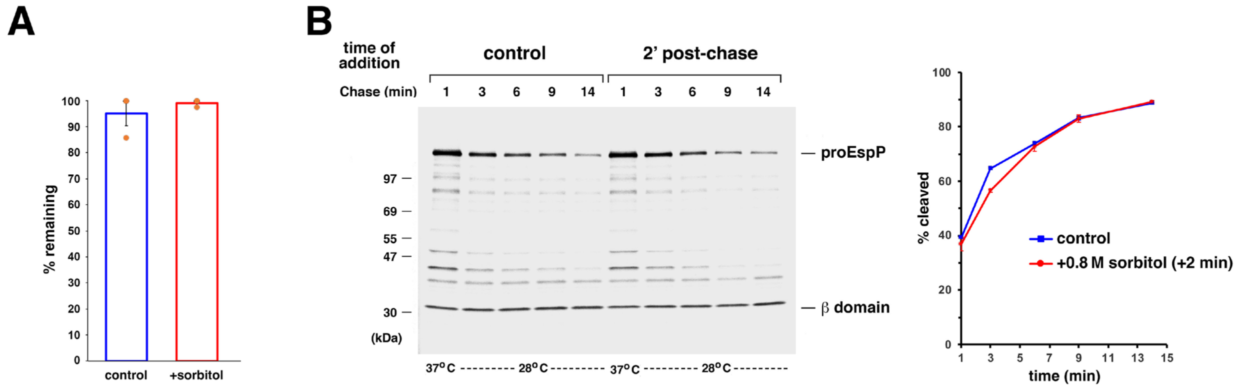

Fig. S23. Sorbitol slightly delays the assembly of wild-type EspP if added after the mutant binds to BAM. (A) The percent of the radiolabeled protein that remained at the 30 min time point in the three independent experiments described in Fig. 6E was calculated as described in the legend to Fig. S20. (B) The experiments described in Fig. 6E were repeated except that AD202 were transformed with pRLS5 instead of pJH224, and after the cells were resuspended in M9 (control) or M9 containing 0.8M sorbitol 2 min after the start of the chase they were incubated in a 28° C shaking water bath for the remainder of the experiment. A gel from a representative experiment is shown on the left and the assembly of EspP in three biologically independent experiments was quantitated on the right by calculating the percent of protein that underwent autocatalytic cleavage. In all experiments data are presented as mean values and error bars represent the SEM. Source data are provided as a Source Data file.

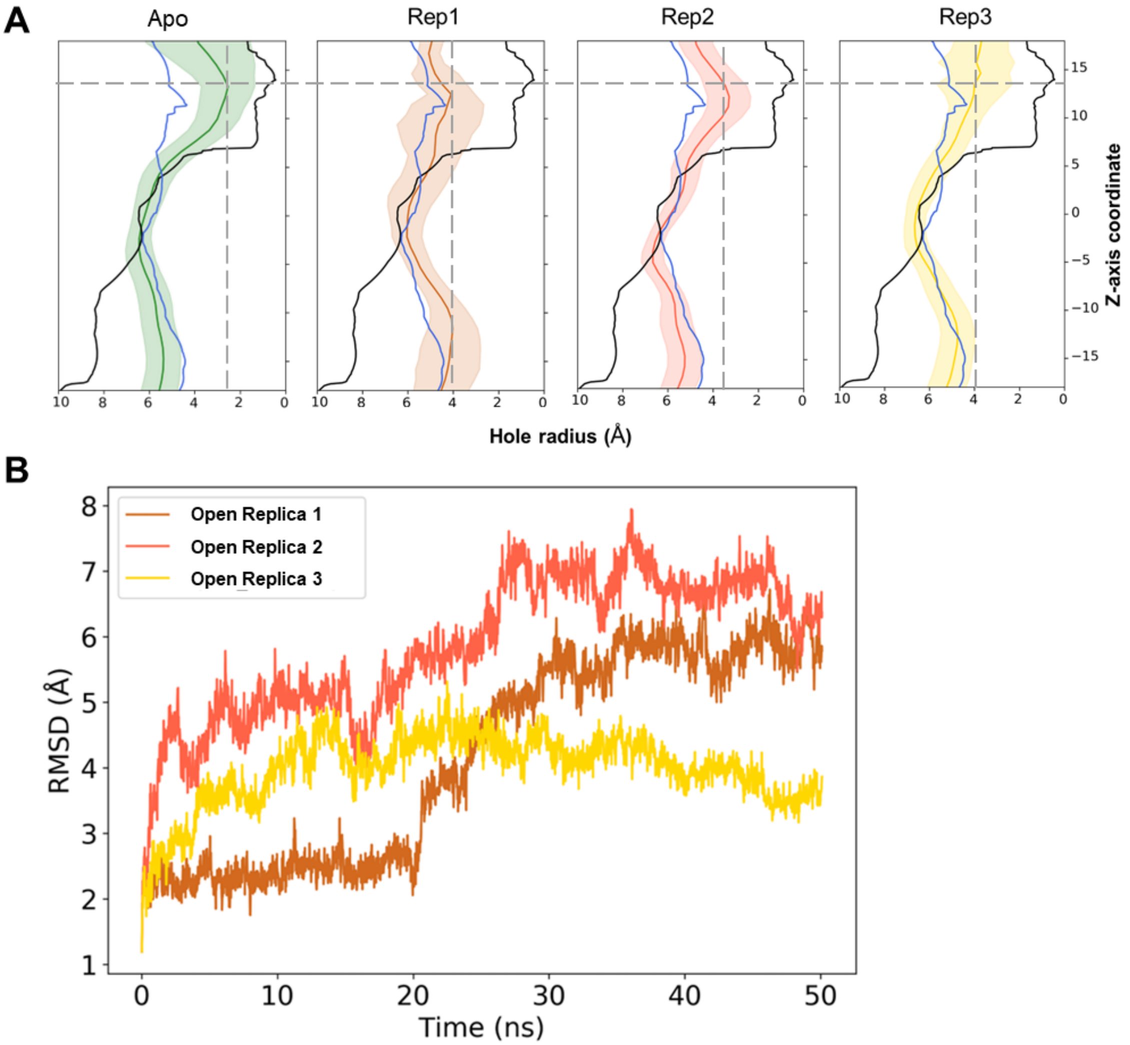

Fig. S24. CP1 does not accelerate BamA β-barrel closure in MD simulations due to docked peptide instability. (A) A hole radius calculation for BamA was performed with the Z coordinate of center of mass (COM) of the membrane set to 0. The hole radius calculation for the closed and open crystal structures is represented by the black and blue lines, respectively. The calculation of hole radius from 50 ns MD simulations for the Apo-Open BamA is shown in green and 3 replicates of open BamA + CP1 are represented in brown, red, and yellow. Dashed gray lines indicate the crosshair of the simulation hole radius at a Z coordinate of 14. (B) The RMSD of lumen binders over the course of the simulations of CP1 bound to the open conformation of BamA. The data in (A) are presented as mean values and error bars represent the standard deviation.

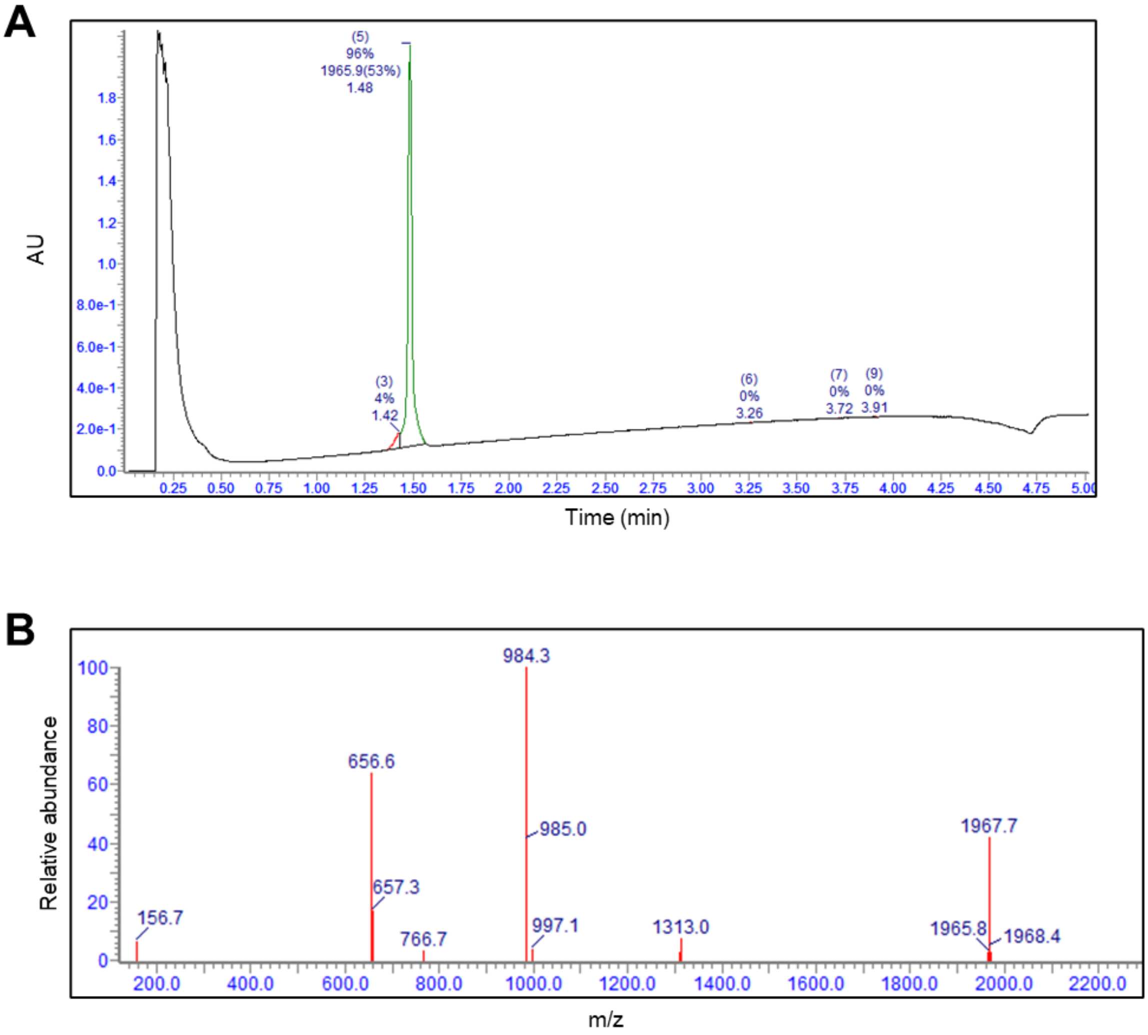

Fig. S25. UPLC/MS analysis of CP1. HPLC trace (A) and mass spectrum of the major peak (B) are shown.

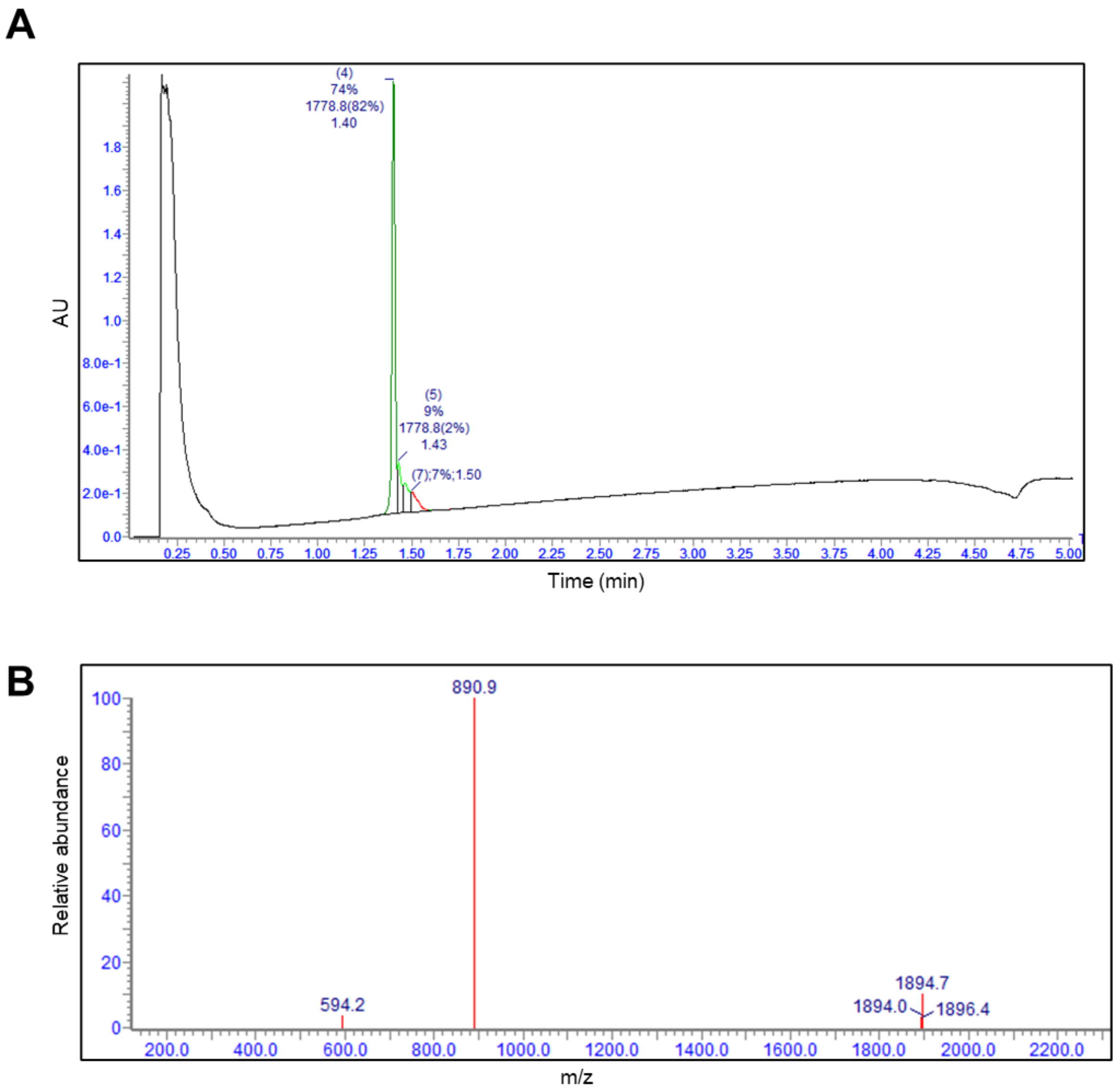

Fig. S26. UPLC/MS analysis of CP2. HPLC trace (A) and mass spectrum of the major peak (B) are shown.

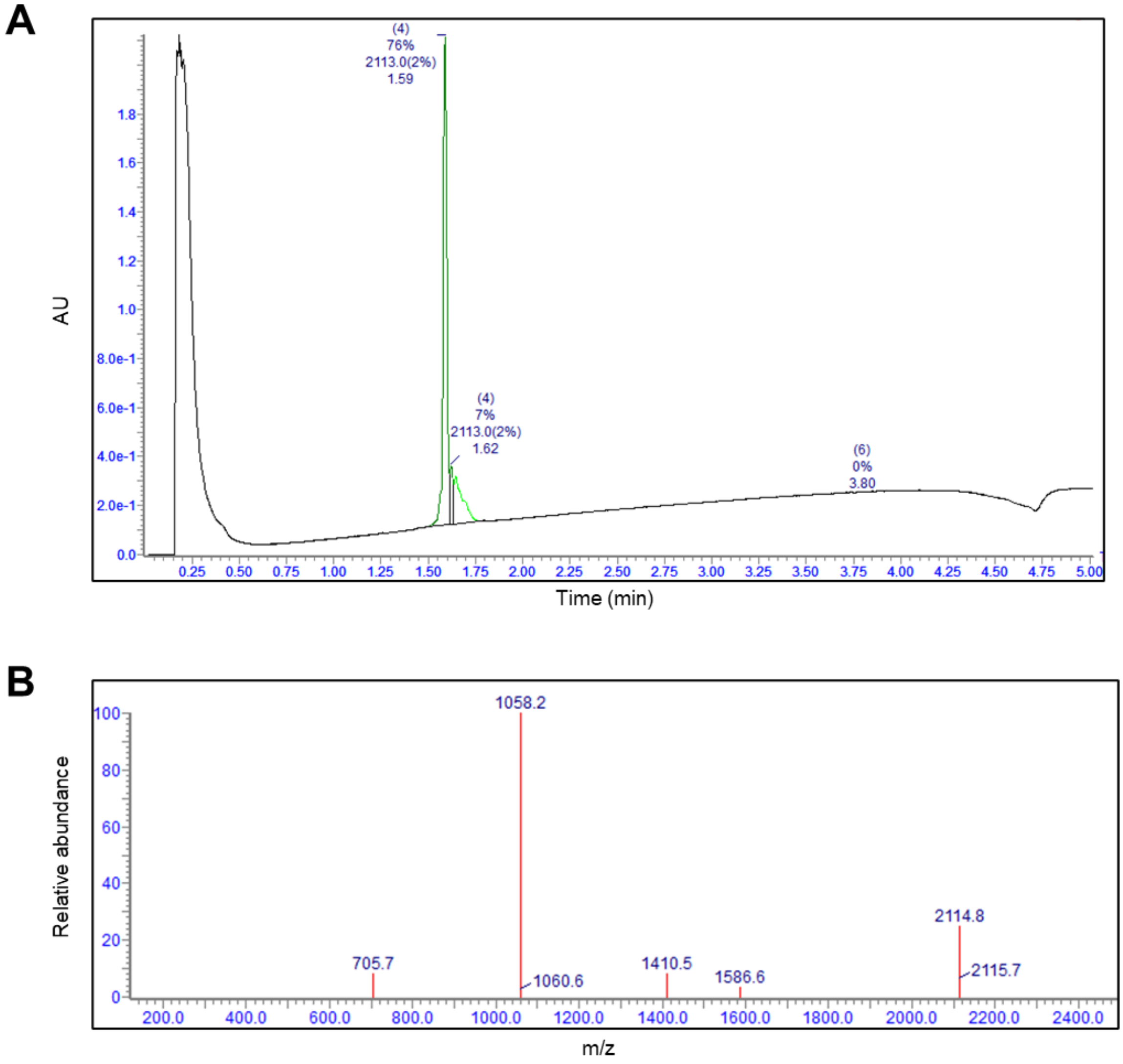

Fig. S27. UPLC/MS analysis of CP3. HPLC trace (A) and mass spectrum of the major peak (B) are shown.

	BamA β-barrel bound to CP1 and Darobactin	BamA β-barrel bound to CP2 and Darobactin	BamA β-barrel bound to CP3
**PDB code**	9CS0	9CS1	9CS2
**Data Collection**			
Wavelength (Å)	1.1807	1.18081	1.00001
Resolution range (Å)	68.952 - 1.944 (2.081 - 1.944)	82.625 - 2.147 (2.286 - 2.147)	78.987 - 1.784 (1.898 - 1.784)
Space group	P 2 2 21	P 2 2 21	P 2 2 21
Unit cell (a, b, c, α, β, γ)	57.954, 81.704, 128.531, 90, 90, 90	58.602, 82.625, 127.889, 90, 90, 90	58.084, 78.987, 124.979, 90, 90, 90
Total reflections	377653 (19006)	229241 (11157)	230051 (8501)
Unique reflections	37294 (1865)	28817 (1442)	45901 (2295)
Multiplicity	10.1 (10.2)	8.0 (7.7)	5.0 (3.7)
Completeness (%)	81.6 (22.4)	83.0 (24.5)	82.6 (24.7)
Mean I/sigma(I)	19.9 (1.4)	24.6 (0.8)	12.4 (1.4)
Wilson B-factor	39.43	52.19	26.45
R-merge	0.075 (1.624)	0.044 (2.165)	0.061 (0.857)
R-meas	0.079(1.709)	0.047 (2.320)	0.068 (1.000)
R-pim	0.025 (0.527)	0.017 (0.819)	0.030 (0.505)
CC1/2	1 (0.607)	1 (0.521)	0.999 (0.511)
**Refinement**			
Resolution range (Å)	64.27 - 1.946 (2.015 - 1.946)	26.64 - 2.147 (2.224 - 2.147)	25.76 - 1.785 (1.848 - 1.785)
Reflections used in refinement	37284 (504)	28786 (538)	45870 (698)
Reflections used for R-free	1799 (27)	1470 (20)	2255 (40)
R-work	0.2305 (0.3184)	0.2262 (0.3820)	0.2156 (0.2708)
R-free	0.2379 (0.3427)	0.2420 (0.4460)	0.2466 (0.2984)
Number of non-hydrogen atoms	3329	3145	3558
macromolecules	3113	3012	3248
ligands	76	65	79
solvent	140	68	231
Protein residues	390	380	401
RMS(bonds)	0.13	0.01	0.159
RMS(angles)	3.28	1.64	3.81
Ramachandran favored (%)	97.31	97.27	97.71
Ramachandran allowed (%)	2.69	2.46	2.29
Ramachandran outliers (%)	0	0.27	0
Rotamer outliers (%)	2.42	3.75	3.74
Clashscore	4.63	6.33	1.74
Average B-factor	48.64	58.85	37.3
macromolecules	48.64	59.1	37.29
ligands	48.68	56.36	32.27
solvent	48.77	50.27	39.23

Table S1. Crystallography statistics.

	7NRE	CP1	CP2	CP3
7NRE	0	0.436	0.422	0.456
CP1	0.436	0	0.246	0.372
CP2	0.422	0.246	0	0.311
CP3	0.456	0.372	0.311	0

Table S2. RMSD values over C-α positions between structures as measured using the align command in PyMOL.

Peptide	Residues
CP1	E435, T461, N463, Y465, Q466, Y468, R488, F494, L581, R592, N594, D614, T615, A616, Y618, L630, R632, T633, R634, Y649, E650, N651, Y653, S657, S658, N666, E717, Q775, P782, E783, V784, N805, T809, W810
CP2	N459, T461, Q466, Y468, E470, S472, T474, S484, L485, G486, G487, R488, F490, N492, F494, D512, T514, L515, G516, S524, L525, R526, G530, V532, T571, N573, G575, W576, T577, T612, D614, R634, Y653

Table S3. Residues in the lumen of the BamA β-barrel located within 4 Å proximity of cyclic peptides in the crystal structures.

Strain	Genotype	Reference
JCM158	MC4100 *ara*$^{r/-}$	[7]
JCM972I	JCM158 *bamA101*	[8]
AM710	JCM158 Δ*bamA* Tn7att::*bamA*FRT *bamB::kan*	[9]
AM711	JCM158 Δ*bamA*Tn7att::*bamA*$_{E470K}$FRT *bamB::kan*	[9]
MB5746	C600 *leu thr lac (thi) galK lpxC (envA1) tolC::tn10*	[10]
HDB164	JCM158 *bamA101 ompT::spc*	this study
AD202	MC4100 *ompT::kan*	[11]

Table S4. *E. coli* strains used in this study.

Compound	Peptide Sequence	Mass (mg)	Yield (%)	Observed m/z	Purity (%)
CP1	F.L.W.MePhe.Y.H.R.P.V.R.G.MePhe.D.C.G	5.2	10	984.3	95
CP2	F.S.G.R.W.Tic.W.P.S.R.S.V.G.C.G	8.9	16	890.9	81
CP3	F.R.R.Y.PeGly.L.D.N.Y.W.V.Tic.Y.C.G	6.1	9	705.7	83

Table S5. Linear peptide sequences and characterization data for compounds CP1, CP2, and CP3. The UPLC/MS data that show the purity and observed m/z are displayed in Figs. S25-S27.

Cycle	Method (CP2 and CP3)	Method (CP1)
15	Double-75°C	Double-50°C
14	Double-50°C	Double-50°C
13	Double-50°C	Double-50°C
12	Double-50°C	Double-50°C
11	Double-50°C	Double-75°C
10	Double-50°C	Double-50°C
9	Double-50°C	Double-50°C
8	Double-50°C	Double-50°C
7	Double-50°C	Double-50°C
6	Double-50°C	Double-50°C
5	Double-50°C	Double-50°C
4	Double-50°C	Double-50°C
3	Single-75°C	Single-75°C
2	Single-50°C	Single-50°C
1	Single-75°C	Single-75°C

Table S6: Coupling conditions used for synthesis of peptides. Cycle number increases from C-terminus to N-terminus (e.g. Cycle 1 is C-terminus, Cycle 15 is N-terminus).

State	PDB	System	Simulation time	Name
Closed	9CS0	Apo (inhibitors removed)	50 ns	Apo_{closed}
	9CS0	CP1	150 ns (50 ns * 3 reps)	$Holo_{CP1\_closed}$
	9CS1	CP2	150 ns (50 ns * 3 reps)	$Holo_{CP2\_closed}$
Open	5LJO	Apo	50 ns	Apo_{open}
		Docked CP1	150 ns (50 ns * 3 reps)	$Holo_{CP1\_open}$
		Docked CP2	150 ns (50 ns * 3 reps)	$Holo_{CP2\_open}$

Table S7. Simulation system information.

SUPPLEMENTARY REFERENCES

1. Terwilliger, T. C. *et al.* Iterative-build OMIT maps: Map improvement by iterative model building and refinement without model bias. *Acta Crystallogr. Sect. D Biol. Crystallogr.* **64**, 515–524 (2008).
2. Afonine, P. V. *et al.* Towards automated crystallographic structure refinement with phenix.refine. *Acta Crystallogr. Sect. D Biol. Crystallogr.* **68**, 352–367 (2012).
3. Liebschner, D. *et al.* Macromolecular structure determination using X-rays, neutrons and electrons: Recent developments in Phenix. *Acta Crystallogr. Sect. D Struct. Biol.* **75**, 861–877 (2019).
4. Luther, A. *et al.* Chimeric peptidomimetic antibiotics against Gram-negative bacteria. *Nature* **576**, 452–458 (2019).
5. Wang, X., Peterson, J. H. & Bernstein, H. D. Bacterial outer membrane proteins are targeted to the bam complex by two parallel mechanisms. *MBio* **12**, (2021).
6. Ieva, R., Tian, P., Peterson, J. H. & Bernstein, H. D. Sequential and spatially restricted interactions of assembly factors with an autotransporter β domain. *Proc. Natl. Acad. Sci. U. S. A.* **108**, (2011).
7. Ricci, D. P., Hagan, C. L., Kahne, D. & Silhavy, T. J. Activation of the Escherichia coli β-barrel assembly machine (Bam) is required for essential components to interact properly with substrate. *Proc. Natl. Acad. Sci. U. S. A.* **109**, 3487–3491 (2012).
8. Aoki, S. K. *et al.* Contact-dependent growth inhibition requires the essential outer membrane protein BamA (YaeT) as the receptor and the inner membrane transport protein AcrB. *Mol. Microbiol.* **70**, 323–340 (2008).
9. Hart, E. M. *et al.* A small-molecule inhibitor of BamA impervious to efflux and the outer membrane permeability barrier. *Proc. Natl. Acad. Sci. U. S. A.* **116**, 21748–21757 (2019).
10. Kodali, S. *et al.* Determination of selectivity and efficacy of fatty acid synthesis inhibitors. *J. Biol. Chem.* **280**, 1669–1677 (2005).
11. Akiyama, Y. & Ito, K. Secy protein, a membrane-embedded secretion factor of E. coli, is cleaved by the ompT protease in vitro. *Biochem. Biophys. Res. Commun.* **167**, 711–715 (1990).
