# Supplementary material for: Antibacterial macrocyclic peptides reveal a distinct mode of BamA inhibition
Source: Nat Commun. 2025 Apr 10;16:3395. doi: 10.1038/s41467-025-58086-w (PMC11986105; doi:10.1038/s41467-025-58086-w)
Supplement: Supplementary file 2 — Reporting Summary [file 41467_2025_58086_MOESM2_ESM.pdf]

nature portfolio

Corresponding author(s): Harris D. Bernstein and Scott S. Walker

Last updated by author(s): Feb 21, 2025

Reporting Summary

Nature Portfolio wishes to improve the reproducibility of the work that we publish. This form provides structure for consistency and transparency in reporting. For further information on Nature Portfolio policies, see our Editorial Policies and the Editorial Policy Checklist.

Statistics

For all statistical analyses, confirm that the following items are present in the figure legend, table legend, main text, or Methods section.

n/a	Confirmed	
☐	☒	The exact sample size (*n*) for each experimental group/condition, given as a discrete number and unit of measurement
☐	☒	A statement on whether measurements were taken from distinct samples or whether the same sample was measured repeatedly
☒	☐	The statistical test(s) used AND whether they are one- or two-sided<br>*Only common tests should be described solely by name; describe more complex techniques in the Methods section.*
☒	☐	A description of all covariates tested
☒	☐	A description of any assumptions or corrections, such as tests of normality and adjustment for multiple comparisons
☐	☒	A full description of the statistical parameters including central tendency (e.g. means) or other basic estimates (e.g. regression coefficient) AND variation (e.g. standard deviation) or associated estimates of uncertainty (e.g. confidence intervals)
☒	☐	For null hypothesis testing, the test statistic (e.g. *F*, *t*, *r*) with confidence intervals, effect sizes, degrees of freedom and *P* value noted<br>*Give P values as exact values whenever suitable.*
☒	☐	For Bayesian analysis, information on the choice of priors and Markov chain Monte Carlo settings
☒	☐	For hierarchical and complex designs, identification of the appropriate level for tests and full reporting of outcomes
☒	☐	Estimates of effect sizes (e.g. Cohen's *d*, Pearson's *r*), indicating how they were calculated

Our web collection on statistics for biologists contains articles on many of the points above.

Software and code

Policy information about availability of computer code

Data collection	For MD simulations, we utilized the following software:<br>Gram-Negative Outer Membrane Modeler (GNOMM) (v 1.0)<br>GROMACS (v 2023.1)<br>Externally published algorithms/models (version unspecified, cited in text): TIP3P water model, SHAKE algorithm, Force-based switching, function, the CHARMM36 force field
Data analysis	BIAcore Evaluation software (Cytiva) was used for SPR data analysis.<br><br>Global Phasing softwares autoPROC with STARANISO (release 20220608), Grade (1.2.19), and Buster (2.11.8) were used for X-ray crystallography data processing, ligand generation, and refinement. CCP4 Dimple (within CCP4 v8.0) was used for phasing and Coot (v0.9.8.1) was used for manual refinement and ligand placing.<br><br>MD simulations were analyzed using the particle-mesh Ewald method for long-range electrostatic interactions (v), the HOLE2 program to calculate the pore profile (v 2.0) , and Matplotlib to produce plots of the average pore radii (v 3.7.1).<br><br>Statistical analysis of outer membrane protein assembly both in live cells and in vitro was performed using Microsoft Excel for Mac v. 16.

For manuscripts utilizing custom algorithms or software that are central to the research but not yet described in published literature, software must be made available to editors and reviewers. We strongly encourage code deposition in a community repository (e.g. GitHub). See the Nature Portfolio guidelines for submitting code & software for further information.

Data

Policy information about availability of data

All manuscripts must include a data availability statement. This statement should provide the following information, where applicable:

- Accession codes, unique identifiers, or web links for publicly available datasets
- A description of any restrictions on data availability
- For clinical datasets or third party data, please ensure that the statement adheres to our policy

The final coordinates of BamA bound to darobactin and CP1, BamA bound to darobactin and CP2, and BamA bound to CP3 have been deposited in the RCSB Protein Data Bank with accession codes 9CS0, 9CS1, and 9CS2, respectively. Source data for all of the experiments performed in this study are provided in the Supplement.

Research involving human participants, their data, or biological material

Policy information about studies with human participants or human data. See also policy information about sex, gender (identity/presentation), and sexual orientation and race, ethnicity and racism.

Reporting on sex and gender	No human participants, data, or biological material were involved in this study.
Reporting on race, ethnicity, or other socially relevant groupings	No human participants, data, or biological material were involved in this study.
Population characteristics	No human participants, data, or biological material were involved in this study.
Recruitment	No human participants, data, or biological material were involved in this study.
Ethics oversight	No human participants, data, or biological material were involved in this study.

Note that full information on the approval of the study protocol must also be provided in the manuscript.

Field-specific reporting

Please select the one below that is the best fit for your research. If you are not sure, read the appropriate sections before making your selection.

☒ Life sciences ☐ Behavioural & social sciences ☐ Ecological, evolutionary & environmental sciences

For a reference copy of the document with all sections, see nature.com/documents/nr-reporting-summary-flat.pdf

Life sciences study design

All studies must disclose on these points even when the disclosure is negative.

Sample size	Sample sizes are described in the Methods section for all outer membrane protein assembly assays and were based on practical considerations. That is, samples were limited to a size that would generate a strong signal but minimize costs and the use of valuable purified proteins and facilitate easy sample manipulation. No sample size calculations were performed for other experiments as the techniques used did not require it.
Data exclusions	No data were excluded from analysis.
Replication	mRNA display screening was performed as a single experiment with a series of six cycles of enrichment. IVTT-electrochemiluminescence validation experiments were performed one time.<br>MIC data, competition SPR data, and P. aeruginosa and A. baumannii SPR data were collected as a single data point.<br>The kinetic constants determined by SPR were the average of 2, 3, or 4 replicates.<br>MD simulations were performed in triplicate. Unless otherwise noted, all outer membrane protein assembly assays were performed in triplicate.<br>All attempts at replication were successful.<br>A few experiments that were only performed to provide additional evidence to support strong conclusions and that yielded compelling results were not replicated.
Randomization	Randomization was not required for any of the experiments performed in the study.
Blinding	Blinding was not required for any of the experiments performed in the study.

Reporting for specific materials, systems and methods

We require information from authors about some types of materials, experimental systems and methods used in many studies. Here, indicate whether each material, system or method listed is relevant to your study. If you are not sure if a list item applies to your research, read the appropriate section before selecting a response.

Materials & experimental systems

n/a	Involved in the study
[ ]	[x] Antibodies
[x]	[ ] Eukaryotic cell lines
[x]	[ ] Palaeontology and archaeology
[x]	[ ] Animals and other organisms
[x]	[ ] Clinical data
[x]	[ ] Dual use research of concern
[x]	[ ] Plants

Methods

n/a	Involved in the study
[x]	[ ] ChIP-seq
[x]	[ ] Flow cytometry
[x]	[ ] MRI-based neuroimaging

Antibodies

Antibodies used	Rabbit polyclonal antisera raised against C-terminal peptides of EspP and OmpC and OmpA loop 4 have been described previously:<br>Yap, M. N. F. & Bernstein, H. D. Mutations in the escherichia coli ribosomal protein L22 selectively suppress the expression of a secreted bacterial virulence factor. J. Bacteriol. 195, 2991–2999 (2013).<br>Hussain, S. & Bernstein, H. D. The Bam complex catalyzes efficient insertion of bacterial outer membrane proteins into membrane vesicles of variable lipid composition. J. Biol. Chem. 293, 2959–2973 (2018).<br>Szabady, R. L., Peterson, J. H., Skillman, K. M. & Bernstein, H. D. An unusual signal peptide facilitates late steps in the biogenesis of a bacterial autotransporter. Proc. Natl. Acad. Sci. U. S. A. 102, 221–226 (2005).<br><br>In immunoprecipitations antisera were used at a dilution of 1:500 (for anti-EspP) or 1:250 (for the other antisera).<br><br>Commercially-available antibodies mouse anti-FLAG M2 antibody (Sigma-Aldrich F1804) and MSD Sulfo-tagged labeled anti-mouse antibody (goat) (MesoScale Discovery R32AC-5) were utilized for electrochemiluminescence assays during mRNA display screening validation.
Validation	The anti-OmpA and OmpC antisera were validated by showing that a signal was detected at the expected molecular weight in extracts derived from wild-type cells but not ompA and ompC knockout strains. The anti-EspP antiserum was validated by showing that a signal was detected at the expected molecular weight in extracts derived from cells that contained an espP expression plasmid but not cells that contained the vector alone.<br><br>Mouse anti-FLAG M2 antibody (Sigma-Aldrich F1804) was assessed for purity by microfluidic gel capillary electrophoresis and for specificity by western blot and chemiluminescence. Validation documents are available from the manufacturer.<br><br>MSD Sulfo-tagged labeled anti-mouse antibody (goat) (MesoScale Discovery R32AC-5) was purified by immunoaffinity chromatography using Mouse IgG coupled to agarose beads followed by solid phase adsorption(s) to remove any unwanted reactivities. Validation documents are available from the manufacturer.

Plants

Seed stocks	N/A
Novel plant genotypes	N/A
Authentication	N/A
